# Giant tidal tails of helium escaping the hot Jupiter HAT-P-32 b

**DOI:** 10.1126/sciadv.adf8736

**Published:** 2023-06-07

**Authors:** Zhoujian Zhang, Caroline V. Morley, Michael Gully-Santiago, Morgan MacLeod, Antonija Oklopčić, Jessica Luna, Quang H. Tran, Joe P. Ninan, Suvrath Mahadevan, Daniel M. Krolikowski, William D. Cochran, Brendan P. Bowler, Michael Endl, Gudmundur Stefánsson, Benjamin M. Tofflemire, Andrew Vanderburg, Gregory R. Zeimann

**Affiliations:** ^1^ Department of Astronomy and Astrophysics, University of California, Santa Cruz, Santa Cruz, CA 95064, USA.; ^2^ Department of Astronomy, The University of Texas at Austin, Austin, TX 78712, USA.; ^3^ Center for Astrophysics, Harvard and Smithsonian, Cambridge, MA 02138, USA.; ^4^ Anton Pannekoek Institute for Astronomy, University of Amsterdam, Amsterdam, Netherlands.; ^5^ Department of Astronomy and Astrophysics, Tata Institute of Fundamental Research, Mumbai, India.; ^6^ Department of Astronomy and Astrophysics, The Pennsylvania State University, University Park, PA 16802, USA.; ^7^ Center for Exoplanets and Habitable Worlds, University Park, PA 16802, USA.; ^8^ ETH-Zürich, Institute for Particle Physics and Astrophysics, Zürich, Switzerland.; ^9^ Steward Observatory, The University of Arizona, 933 N. Cherry Ave, Tucson, AZ 85721, USA.; ^10^ Center for Planetary Systems Habitability and McDonald Observatory, The University of Texas at Austin, Austin, TX 78730, USA.; ^11^ McDonald Observatory and the Department of Astronomy, The University of Texas at Austin, Austin, TX 78712, USA.; ^12^ Department of Astrophysical Sciences, Princeton University, Princeton, NJ 08544, USA.; ^13^ Department of Physics and Kavli Institute for Astrophysics and Space Research, MIT, Cambridge, MA 02139, USA.; ^14^ Hobby-Eberly Telescope, The University of Texas at Austin, Austin, TX 78712, USA.

## Abstract

Capturing planets in the act of losing their atmospheres provides rare opportunities to probe their evolution history. This analysis has been enabled by observations of the helium triplet at 10,833 angstrom, but past studies have focused on the narrow time window right around the planet’s optical transit. We monitored the hot Jupiter HAT-P-32 b using high-resolution spectroscopy from the Hobby-Eberly Telescope covering the planet’s full orbit. We detected helium escaping HAT-P-32 b at a 14σ significance,with extended leading and trailing tails spanning a projected length over 53 times the planet’s radius. These tails are among the largest known structures associated with an exoplanet. We interpret our observations using three-dimensional hydrodynamic simulations, which predict Roche Lobe overflow with extended tails along the planet’s orbital path.

## Teaser

Long-baseline monitoring of the HAT-P-32Ab system reveals helium escaping through tidal tails 50 times the size of the planet.

## INTRODUCTION

Atmospheric escape is the primary physical process sculpting the population of short-period, irradiated exoplanets. One piece of observational evidence of this process is the observed dearth of short-period Neptune-mass planets (*1*). Several mechanisms likely contribute to the atmospheric escape, including photoevaporation and core-powered mass loss (*2*–*9*), which predict distinct correlations between mass-loss rates and properties of the radiation environment [e.g., x-ray and ultraviolet (UV) fluxes of host stars] and planets (e.g., equilibrium temperatures). Directly measuring mass loss for a large ensemble of exoplanets can differentiate between these processes, and these measurements have been enabled by the helium 10,833-Å triplet (*10*–*12*), which is a robust probe of exospheres accessible from the ground and immune to high absorption from the interstellar medium that hampers similar studies based on Lyman α.

Using the Habitable-zone Planet Finder Spectrograph (HPF) (*13*–*15*) on the Hobby-Eberly Telescope (HET) (*16*–*18*), we observed HAT-P-32 b with time-series high-resolution (*R* ≈ 55,000) spectra to detect a helium outflow and investigate atmospheric escape. HAT-P-32 b is a hot Jupiter transiting a late-F star, HAT-P-32 A, on a 2.15-day orbit with an in-transit duration of 3.12 hours (*19*, *20*). This planet has an inflated radius (1.79 ± 0.03 Jupiter radii) that almost fills its Roche lobe. A recent study (*21*) found hydrogen and helium outflows escaping HAT-P-32 b using the Calar Alto high-Resolution search for M dwarfs with Exoearths with Near-infrared and optical Échelle Spectrographs (CARMENES) (*22*) on the Calar Alto 3.5-m telescope. They collected high-resolution (*R* ≈ 80,000) spectra of the host star over two nights right around the planet’s optical transits and monitored for 6 hours each night centered on the middle of transits, leading to the detection of the planet’s excess helium absorption with a maximum transit depth of 5.3%.

With HET/HPF, we monitored HAT-P-32 Ab with time-series spectroscopy, covering orbital phases spanning the planet’s full orbital period. We collected spectra during three planet transits on 9 August 2020 universal time (UT), 19 September 2020 UT, and 6 October 2020 UT and during out-of-transit periods within 2 days of each transit. We also monitored the stellar activity of HAT-P-32 A with irregular cadence from 1 August 2020 UT to 25 December 2020 UT (Table 1). Our data were reduced using the HPF pipeline code Goldilocks and the muler Python package (*23*–*26*), which perform bias and nonlinearity corrections, cosmic-ray rejection, flat fielding, wavelength calibration, and careful subtraction of sky emission features. We obtained a total of 77 high-quality spectra with a median signal-to-noise ratio (S/N) of 85 per pixel (142 per resolution element) near 10,833 Å. All spectra were shifted to the stellar rest frame based on barycentric corrections and our computed absolute radial velocities (RVs).

**Table 1.** The HET/HPF observing log of HAT-P-32 Ab. For each date, we list the number of the observed spectra (*N*_spec_) and their mean S/N (per pixel) near 10,833 Å, as well as the range of air mass and orbital phase covered by these data. Each spectrum was acquired with an exposure time of 820 s. The HPF spectral resolution element contains a median of 2.8 pixels. We also divide our spectra into five subsets based on their orbital phase as indicated in the “Type” column, including start (with the orbital phase in [−0.5, −0.15]), pre ([−0.15, −0.03]), transit ([−0.03, +0.03]), post ([+0.03, +0.08]), and end ([+0.08, +0.5]).

Date (UT)	Type	*N*_spec_	Mean S/N	Air mass	Orbital phase
Transit event 1					
7 August 2020	post	3	57	[1.31, 1.20]	[+0.05, +0.06]
8 August 2020	start	4	91	[1.30, 1.19]	[−0.49, −0.48]
9 August 2020	transit	6	68	[1.36, 1.16]	[−0.03, −0.01]
Transit event 2					
18 September 2020	start	3	89	[1.14, 1.20]	[−0.37, −0.36]
19 September 2020	transit	6	84	[1.35, 1.16]	[−0.01, +0.01]
19 September 2020	end	2	112	[1.16, 1.18]	+0.10
20 September 2020	start	4	93	[1.15, 1.24]	[−0.44, −0.43]
22 September 2020	end + start	4	95	[1.15, 1.24]	[+0.49, +0.51]
Transit event 3					
5 October 2020	end	4	96	[1.29, 1.18]	[+0.42, +0.43]
6 October 2020	pre	6	74	[1.31, 1.14]	[−0.12, −0.10]
6 October 2020	transit	6	93	[1.14, 1.31]	[−0.02, 000]
7 October 2020	end	4	96	[1.15, 1.24]	[+0.45, +0.46]
8 October 2020	start	4	83	[1.29, 1.18]	[−0.19, −0.18]
Stellar activity monitoring					
1 August 2020	end	2	99	[1.21, 1.18]	+0.28
4 September 2020	post	4	59	[1.31, 1.19]	[+0.04, +0.05]
5 September 2020	start	1	84	1.17	−0.48
11 October 2020	end	2	116	[1.18, 1.21]	+0.31
12 October 2020	start	4	100	[1.17, 1.27]	[−0.23, −0.22]
2 December 2020	end	2	66	[1.30, 1.26]	+0.32
9 December 2020	start	2	90	[1.17, 1.20]	[−0.33, −0.32]
23 December 2020	end	2	71	[1.20, 1.23]	+0.17
25 December 2020	end	2	74	[1.17, 1.20]	[+0.09, +0.10]

## RESULTS

**Fig. 1.**
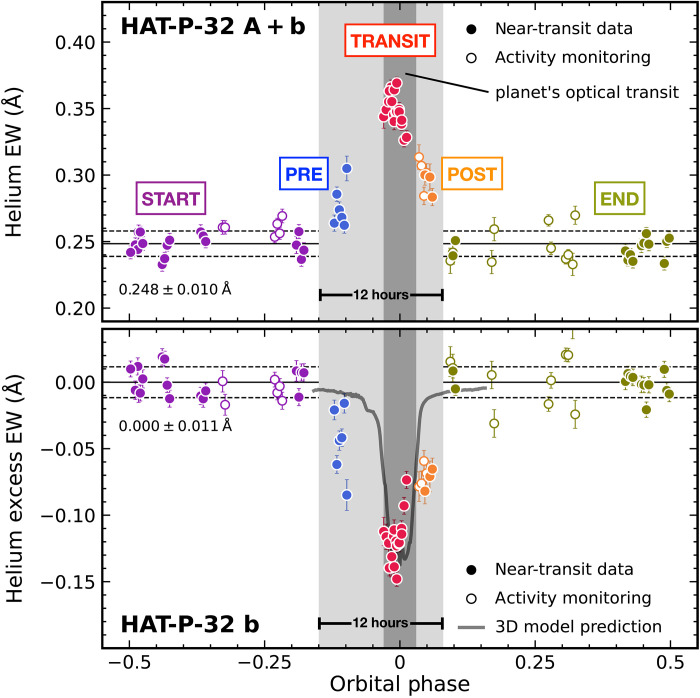
Measured helium excess in HAT-P-32 A+b planetary system. EWs of the helium 10,833-Å triplet, measured from the observed spectra of HAT-P-32 A + b (top) and computed residual spectra of HAT-P-32 b (bottom), exhibit long-duration (12 hours), significant (14σ) excess near the planet’s transits. Solid circles represent data obtained within two nights of each optical transit, and open circles represent the stellar variability monitoring data obtained from out-of-transit periods. We divided the data into five categories in terms of orbital phase, with boundaries highlighted by gray-shaded regions. Our three-dimensional (3D) hydrodynamic simulation is shown as the gray solid line. The time-series helium EWs are asymmetric with respect to the planet’s optical transit (dark gray), demonstrating the leading tail of the helium atmosphere escaping HAT-P-32 b.

We measured helium equivalent widths (EWs) for all HPF spectra of HAT-P-32 A + b and detected a long-duration (12 hours), statistically significant (14σ) excess absorption feature near the transit of HAT-P-32 b (Fig. 1). The helium excess is not correlated with any stellar activity indicators (fig. S1) and spans ≈4× longer than the planet’s optical transit duration. To facilitate the transmission spectroscopic analysis, we divided the data into five subsets according to their orbital phases ϕ (Fig. 1), which we denote as start (−0.5 ≤ ϕ ≤ −0.15; 23 spectra), pre (−0.15 < ϕ ≤ −0.03; 6 spectra), transit (−0.03 < ϕ < +0.03; 18 spectra), post (+0.03 ≤ ϕ ≤ +0.08; 7 spectra), and end (+0.08 < ϕ ≤ +05; 23 spectra). We constructed the out-of-transit reference spectra for each of the three transits by combining the corresponding start and/or end spectra (fig. S2) and used them to normalize the other data to produce transmission and residual (=transmission − 1) spectra.

**Fig. 2.**
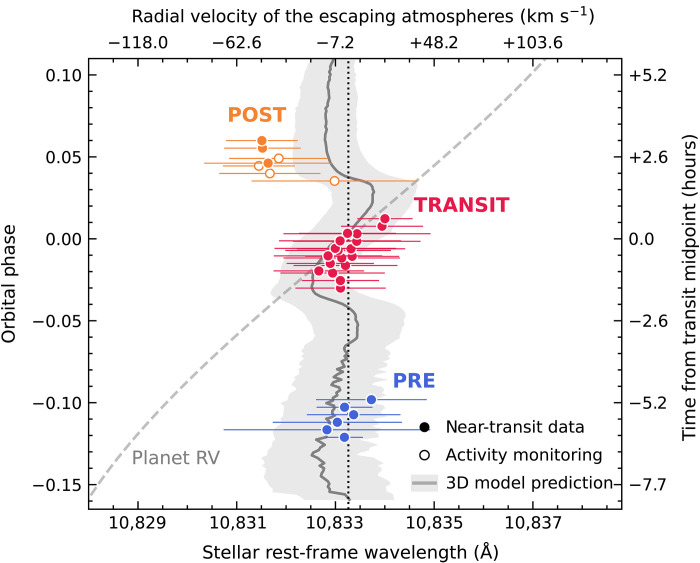
Measured central wavelengths and RVs of HAT-P-32 b’s escaping helium atmosphere as a function of orbital phase in the stellar rest frame. The observed helium excess features in pre (blue), transit (red), and post (orange) residual spectra were fitted by Gaussian profiles to determine the central wavelengths and the standard deviations (circles with horizontal bars), which were then converted to RVs of the escaping atmosphere based on the rest wavelength of the two strongest components of the helium triplet (vertical dotted line at 10,833.26 Å). Circle symbols have the same format as those shown in Fig. 1. The gray dashed line represents the planet’s RV. The central wavelength and SD of helium excess from our simulations are shown as the gray solid line with shadow.

We detected strong excess helium absorption from pre, transit, and post residual spectra and measured the time-dependent wavelength shift for each subset to characterize the velocity of HAT-P-32 b’s escaping atmosphere (Fig. 2). We modeled the excess helium absorption feature in each residual spectrum (figs. S3 and S4) using a Gaussian profile to determine its transit depth and central wavelength, and then we converted the latter into an RV of the escaping atmosphere (in the stellar rest frame) by comparing with the rest wavelength of the helium triplet. As shown in Fig. 2, RVs of the helium excess during the optical transits of HAT-P-32 b are consistent with those of the planet’s orbital motion, reaffirming the planetary origin of the helium; the observed features have slightly higher RVs (i.e., toward the star) than the planet’s orbital RV, which could result from mass transfer from the planet to its host star. These observations are consistent with both Roche lobe overflow and mass loss controlled by planetary and stellar magnetic fields [e.g., (*27*)]. Before (pre) and after (post) the planet’s optical transit, the observed escaping atmosphere does not track the planet’s orbital motion and, instead, has only a small line-of-sight velocity shift in the stellar rest frame, suggesting that the helium gas from the planet’s upper atmosphere mostly moves within the sky plane perpendicular to the observers’ line of sight; this property is consistent with the spatially extended geometry of the gas (as indicated by the long duration of our detected helium excess; see Fig. 1), with gas far from the planet orbiting the star. Helium excess features in post residual spectra are noticeably blue-shifted, implying that material trailing the planet is moving outward in the planetary system.

**Fig. 3.**
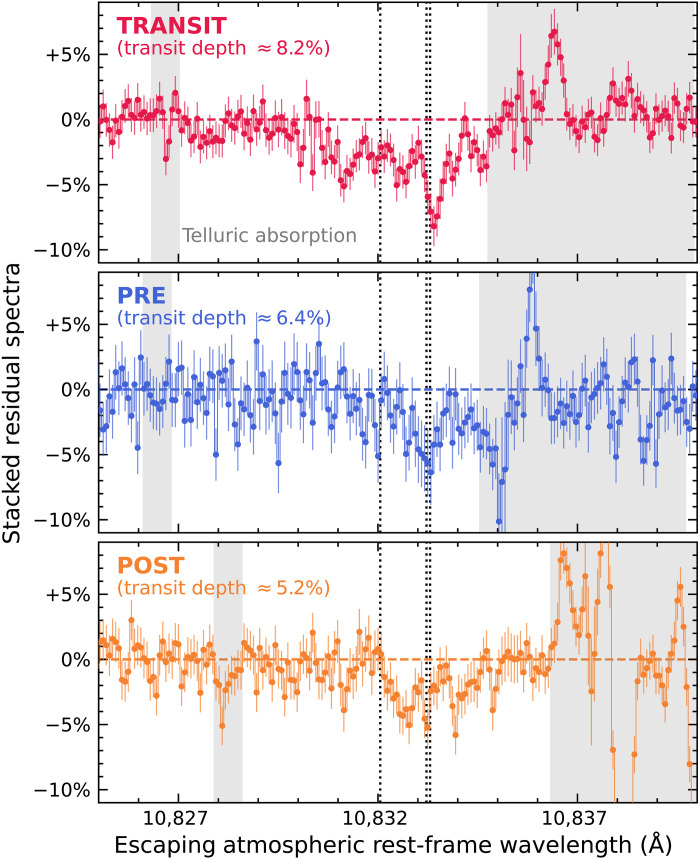
Stacked residual spectra of HAT-P-32 b. Before the stacking, each residual spectrum has been corrected by its RV of the escaping helium atmosphere, such that their helium excess features all line up with the rest wavelength of the strongest component of the helium triplet. We detected strong excess helium absorption from transit (top), pre (middle), and post (bottom) residual spectra, spanning 1.5 to 4 Å in wavelength with a maximum depth of 8.2%, 6.4%, and 5.2%, respectively. The rest wavelength of the helium triplet is marked by vertical dotted lines. Telluric absorption features with a transmission of <99.9% are shown as gray shades.

The maximum depth of the detected helium excess is measured to be ≈8.2% during the optical transit of HAT-P-32 b; the helium excess in the 12-hour period surrounding optical transit is 5 to 6% (Fig. 3). Our in-transit helium excess depth is about 1.5 times higher than the value (5.3%) measured by CARMENES because their “out-of-transit data” were taken when the escaping helium is still in transit. Our detected excess helium absorption spans 3 to 4 Å in the stacked transit residual spectrum, comparable with the observed residual spectra from CARMENES (*21*), and spans 1.5 to 2 Å in the stacked pre and post residual spectra. While other planets show trailing tails of material (*12*, *28*–*30*), HAT-P-32 b has both a (longer) leading and (shorter) trailing tail.

## DISCUSSION

### Helium outflow from three-dimensional hydrodynamic models

**Fig. 4.**
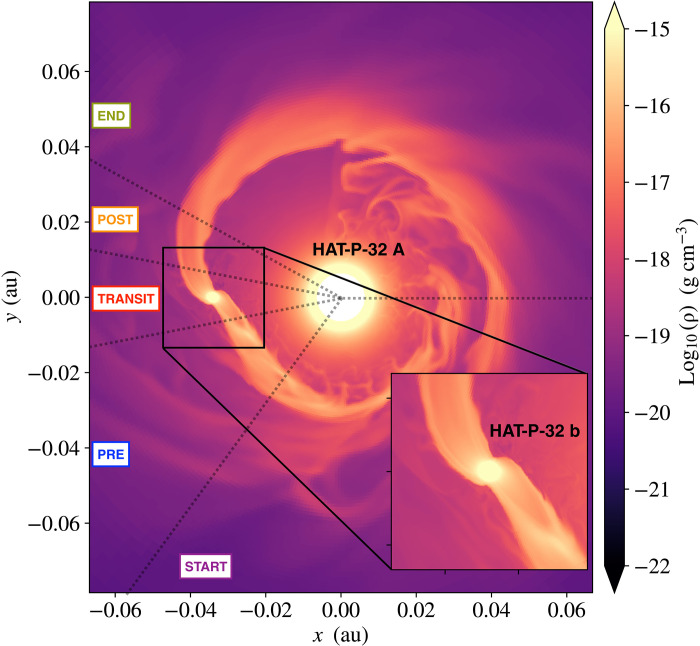
Slice through the orbital plane of a simulated system approximating HAT-P-32 A + b. The frame rotates with the planetary mean motion, so the position of observers rotates clockwise, with regions of start, pre, transit, post, and end shown in observations divided by black dotted lines. The logarithm of gas density is shown in the color scale. A low-density but relatively fast stellar wind expands from HAT-P-32 A at the coordinate origin and interacts with the outflow from HAT-P-32 b. The outflow from HAT-P-32 b is stretched into long, column-like tails leading and trailing the planet along the orbital path. These tidal tails are shaped by the advection of slow-moving planetary outflow in the star-planet gravitational field.

Comparing our observations to three-dimensional (3D) hydrodynamic simulations of the HAT-P-32 A + b system provides physical insight into the geometry of the outflow. We generated 3D hydrodynamic models following (*31*) to examine the interactions between the planetary outflow and stellar winds in the tidal gravitational field of the HAT-P-32 A + b system (Fig. 4). Because of the small orbital separation and high star-to-planet mass ratio, HAT-P-32b nearly fills its Roche lobe. Our models show extended, columnar tails of planetary outflow both leading and trailing the planet along the orbital path. These tails provide excess helium absorption even at phases far from the planet’s optical transit that match our observations. Our model also predicted that the mass loss rate of the planet is ≈1.7 × 10^−14^ *M*_⊙_ year^−1^, implying that the planet will lose its atmosphere over a time scale of $M_{p}/{\dot{M}}_{p}\approx 4\times 10^{10}$ years.

As shown in Fig. 1, our 3D model does not accurately explain the observed relative depth of the pre and post phases as compared to the mid-transit, which we used as a point of reference. However, as traced in Fig. 2, the model predicted that the extended tidal tails lie in approximately the stellar rest frame, not the planet’s rest frame, as seen in our HPF data. Our simulations (Figs. 2 and 4) reveal how these extended tails span the star-planet environment of the HAT-P-32 A + b system. Sophisticated models that account for the momentum deposition by the stellar radiation field on the planetary outflow, orbital eccentricity of the planet, ram pressure of stellar wind, and the thermodynamics of the planetary outflow will be useful to probe the range of physical processes shaping this interaction.

### The exceptional escaping helium of HAT-P-32 b

HAT-P-32 b’s escaping helium atmosphere is exceptional among known detections: It has the largest depth and longest duration found to date. The duration of the helium transit implies the tidal tails have a sky-projected length of 53 times the planet’s radius (seven times the host star’s radius), among the largest structures ever observed in a planetary system. Our study verifies the importance of long-baseline monitoring of planet-host systems to characterize systems with extended tails. Many surveys have targeted K-type planet-host stars since their UV spectra can readily populate the helium metastable state (*32*); HAT-P-32 Ab’s strong helium excess empirically demonstrates that F stars also provide a suitable environment for mechanisms triggering the planets’ mass loss. Our observations of HAT-P-32 b show that this planet-star configuration, where the planet largely fills its Roche lobe, can lead to extended outflowing material.

### On the potential variability of HAT-P-32 b’s helium excess

**Fig. 5.**
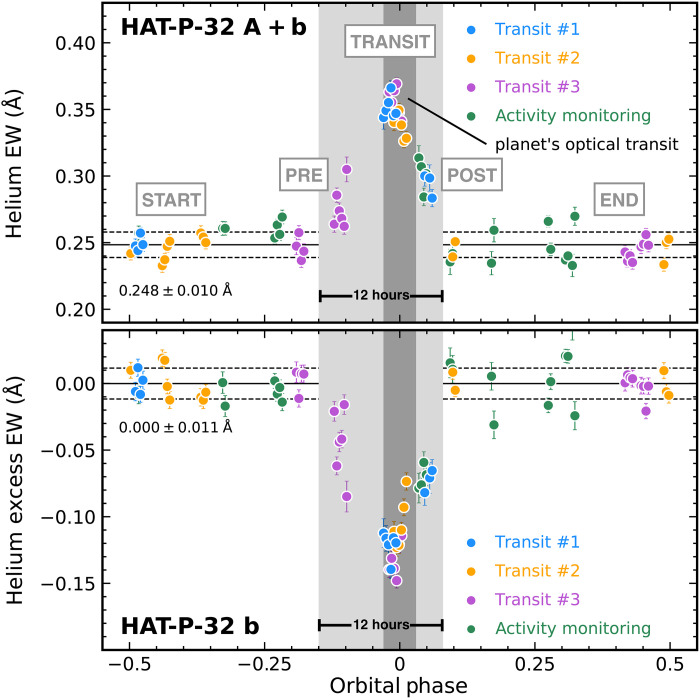
The measured helium EWs of HAT-P-32 A + b and the helium excess EWs of HAT-P-32 b from different dates are consistent over a given range of the planet’s orbital phase. The data presented here are exactly the same as Fig. 1 but are color-coded by observation dates, including those observed during the first (blue), second (orange), and third (purple) transit event, as well as the long-term stellar activity monitoring (green). More observations slightly before the planet’s optical transit are needed to examine whether the planet’s helium excess is variable in the pre subset.

We examined the potential variability of HAT-P-32 b’s escaping helium atmosphere by comparing measured helium excess EWs across different dates (Fig. 5). During the planet’s optical transit, the helium excess EWs mostly varied by less than 0.03 Å across three events. Near the planet’s egress (observed during the second transit event) and slightly after the optical transit (shown by the post subset), EWs of helium excess varied by less than 0.02 Å. In the pre subset, all our observations were collected on the same night with the third transit event. In this subset, the measured helium excess EWs varied by 0.06 Å, and the helium excess absorption appears to become stronger with the increasing orbital phase. More observations of HAT-P-32 A + b over the orbital phase of the pre subset will be useful to assess the variability of the planetary helium outflow slightly before the optical transit. To conclude, these HPF observations do not suggest a significant variability of HAT-P-32 b’s helium excess over our monitoring baseline from 7 August 2020 to 25 December 2020. As a point of comparison, in our 3D hydrodynamic simulations, the overall column density of metastable helium and its mean RV do not appear significantly variable despite instabilities at the interface between the planetary and stellar winds.

Comparing the helium excess revealed by the HPF spectra with those from the CARMENES data (*21*), observed on 1 September 2018 and 9 December 2018, will examine the longer-term variability of the planet’s helium excess. The published CARMENES spectroscopic observations span from about 4 hours before (≈−0.08 in orbital phase) to 3 hours after (≈+0.06 in orbital phase) the midpoint of the planet’s optical transit, meaning they coincide with the pre, transit, and post subsets in our analysis (Fig. 1). New CARMENES observations that cover a longer time baseline (especially with orbital phases of our start and end subsets) than those already acquired by (*21*) will construct the reference spectra that are needed to reanalyze the CARMENES-based helium excess. Comparing these results with the HPF measurements will investigate the long-term variability for HAT-P-32 b’s outflow.

### The diversity of planetary systems with escaping helium atmospheres

**Fig. 6.**
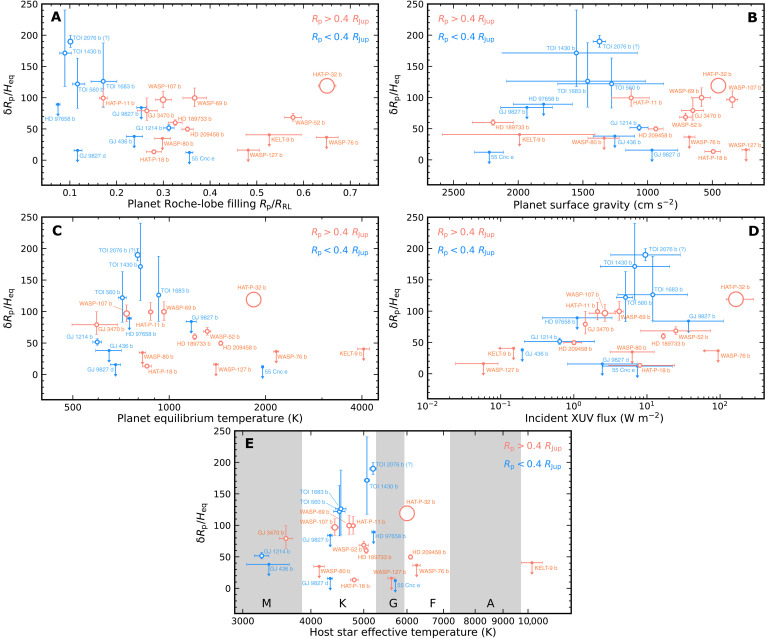
Census of planetary systems with detections and upper-limit constraints of excess helium absorption (table S1), divided into gas giants (orange) and sub-Neptunes (blue). The *y* axes of all panels present the objects’ equivalent heights of helium upper atmosphere in units of the scale height at equilibrium temperatures, δ*R*_p_/*H*_eq_. The *x* axis in (**A**) is Roche-lobe filling *R*_p_/*R*_RL_, which is the radius ratio between the planet and its Roche lobe. The *x* axes in (**B** to **E**) represent these planets’ surface gravities, equilibrium temperatures, incident XUV flux from their host stars, and their host stars’ effective temperatures, respectively. Symbol size for detections is proportional to the ratio between the transit duration of helium excess and that of the planets’ optical transits. Most systems have such ratios as 1, while WASP-69 b (*12*), WASP-107 b (*28*), and HAT-P-32 b have larger ratios of 1.2, 1.4, and 3.8, respectively, meaning that their helium upper atmospheres are extended. In addition, TOI 2076 b exhibited excess helium absorption until 50 min after the planet’s egress; we computed a ratio of 1.3 although the monitoring baseline did not extend the full optical transit and preingress periods (*60*). Furthermore, Gaidos *et al.* (*61*) recently cautioned that the excess helium absorption signature of TOI 2076 b is likely due to its host star’s variability. In addition, TOI 1430 b exhibits excess helium absorption slightly before the planet’s ingress (*60*); longer-baseline monitoring would validate this feature so we simply assumed a ratio of 1.

We compared properties of HAT-P-32 Ab with all other planetary systems that have either detections or upper-limit constraints of the escaping helium atmospheres, to investigate what physical parameters are causing the unusually extended helium atmosphere of HAT-P-32 b and driving the mass loss of exoplanets in general. We compiled all these systems in table S1. Following (*12*), we computed the equivalent height of each planet’s helium atmosphere δ*R*_p_ and its ratio to the planet’s atmospheric scale height at the equilibrium temperature *H*_eq_, leading to a metric, δ*R*_p_/*H*_eq_, that quantifies the strength of the helium excess signal [also see (*33*–*35*)]. The census was divided into two subsets with planetary radii above and below 0.4 *R*_Jup_, which represent gas giants and sub-Neptunes, respectively. In Fig. 6, we investigated δ*R*_p_/*H*_eq_ as functions of the planets’ Roche-lobe filling, planetary surface gravity, the planet’s bolometric equilibrium temperature, incident x-ray and UV (XUV) flux from the host stars, and the host stars’ effective temperatures. Here, the Roche-lobe filling stands for the ratio between the planetary radii *R*_p_ to the Roche lobe radii *R*_RL_, with the latter computed via equation 2 of (*36*).

With the existing census, Fig. 6 does not suggest clear trends between δ*R*_p_/*H*_eq_ and other physical properties investigated here. Among gas giants, HAT-P-32 b has the largest δ*R*_p_/*H*_eq_ and the longest-duration helium excess. This property lines up with its much larger Roche-lobe filling, lower surface gravity, and higher XUV incident flux, all of which are expected to foster the planet mass loss. However, several planets (e.g., WASP-69 b and WASP-107 b) with lower Roche-lobe fillings and XUV incident fluxes achieved comparable δ*R*_p_/*H*_eq_ as HAT-P-32 b. In addition, WASP-76 b has very similar properties as HAT-P-32 b, but its potential helium outflow was not significantly detected as shown by (*37*), although partially due to the contamination of the telluric absorption near the helium triplet.

Note that the three planets with the deepest helium excess depths, WASP-69 b, WASP-107 b, and HAT-P-32 b, also have longer-duration extended excess absorption than the planets’ optical transits. A variety of system physical properties must conspire to create extended, deep helium signals; surface gravity—and therefore escape velocity—seems to be important since these three planets have some of the lowest in the sample. The observed diversity calls for further observational and theoretical studies of planets with both detections and nondetections of excess helium absorption with long-baseline monitoring, to probe the mass loss mechanisms of exoplanets.

## MATERIALS AND METHODS

### Known properties of the HAT-P-32 system

Located at a distance of 283 ± 2 pc (*38*), HAT-P-32 A is a moderately rapidly rotating (*v* sin *i* = 20.7 ± 0.5 km s^−1^) late-F dwarf with a near-solar metallicity ([Fe/H]= −0.04 ± 0.08 dex) and an isochrone-based age of 2 to 4 Ga [e.g., (*19*, *39*)]. The hot Jupiter HAT-P-32 b was found by Hartman *et al.* (*19*) from optical transit light curves and the host star’s multiepoch RVs. The observed RV jitter (≈80 m s^−1^) of HAT-P-32 A prevents precise measurements of the planet’s orbital eccentricity, so Hartman *et al.* (*19*) provided two sets of orbital solutions for HAT-P-32 b by assuming a circular orbit (i.e., *e* = 0) or allowing the eccentricity to vary (leading to *e* = 0.163 ± 0.061). On the basis of new RV data and/or transit light curves, Wang *et al.* (*20*) and Knutson *et al.* (*40*) refined the planet’s orbital eccentricity as $e=0.159_{-0.028}^{+0.051}$ and $e=0.20_{-0.13}^{+0.19}$, respectively. However, the secondary eclipse timing of the planet detected by Zhao *et al.* (*41*) clearly suggests a lower $e=0.007_{-0.006}^{+0.070}$. Throughout this work, we thus adopt a circular planet orbit for HAT-P-32 b. Various orbital analyses converge on the planet’s orbital semi-major axis of ≈0.034 arbitrary units (au) (*19*, *20*, *41*). HAT-P-32 b has a mass of 0.59 ± 0.03 *M*_Jup_, a radius of 1.79 ± 0.03 *R*_Jup_, a low density of 0.14 ± 0.02 g cm^−3^, and a high equilibrium temperature of 1836 ± 7 K (*19*, *21*). With HAT-P-32 b’s mass and irradiation, a core-less planet (the limiting case that provides the highest possible radius) would have a radius of 1.1 to 1.2 *R*_Jup_ [e.g., (*42*)], meaning that HAT-P-32 b is an inflated hot Jupiter. This planet likely has a polar orbit, with a sky-projected obliquity of 85.0° ± 1.5°, based on the Rossiter-McLaughlin effect (*43*). The HAT-P-32 A + b system also has an M1.5 stellar companion, HAT-P-32 B, with a projected separation of 2.94″ or 830 au (*41*, *44*, *45*). With such a wide orbit, HAT-P-32 B is not responsible for the detected significant RV trend (−38 ± 5 m s^−1^ year^−1^) of HAT-P-32 A (*20*, *40*).

### HET/HPF observations, data reduction, and postprocessing

HET is designed with a fixed elevation of 55° and a tracking window of ±8° (*16*, *17*). As a consequence, HAT-P-32 (declination = 46.687852°) is only observable over a track length of 1.5 to 3.3 hours per night. The strategy of our survey is to observe the target over the full track when the planet’s optical transit occurs and to collect out-of-transit spectra on both of the two nights before and two nights after the transit event to construct the reference spectra used to calibrate the transmission spectra. We also monitored HAT-P-32 A + b from 1 August 2020 UT to 25 December 2020 UT with irregular cadence to investigate its stellar activity. Our observing log is summarized in Table 1.

Our raw HPF data were reduced using the Goldilocks pipeline (*23*–*25*), which extracts the 1D simultaneous spectra from the target fiber, sky reference fiber, and the laser frequency comb (LFC) fiber from 2D images, spanning from 8100 to 12,800 Å. Given the relaxed requirement about the RV precision for the science goal of our survey and to avoid any scattered light in the target star fiber, the LFC was turned off. We applied standard 1D postprocessing for these spectra using our newly developed muler framework (*26*) that also provides quick-look quality assurance.

To remove sky emission lines, we combined the observed spectra from both the target and the sky fibers. These two types of fibers have different throughputs, so the direct/naive subtraction between target and sky fiber spectra is not sufficient. The sky-to-target throughput calibration was determined by our muler framework. Specifically, we analyzed twilight flats regularly acquired during the HPF operations. In this configuration, both target and sky fibers were illuminated by the scattered sunlight. Computing the ratio of the solar spectra acquired by these two fibers, we thus derived a wavelength-dependent scaling factor that should be applied to the sky fiber before the sky subtraction. We found the sky fiber has a ≈7% more throughput than the target fiber. We further validated this sky-to-target throughput calibration using another dataset acquired during the HPF nighttime operations. In this dataset, both target and sky fibers were pointed at blank patches of the sky, leading to two sets of spectra populated with only sky emission lines. Comparing the strengths of these skylines from both fibers led to a similar calibration term determined from the twilight flats (also see the documentation and tutorials of muler). Overall, the sky subtraction based on muler leads to 14 improvements over the naive sky subtraction, with negligible residual structure seen in most bright skylines near the helium triplet. The performance of our sky subtraction is also verified by another independent method that can derive the sky-to-target throughput calibration using a given target’s observed spectrum without requiring twilight flats or blank sky observations (*46*). As an additional step to further minimize the impact of skylines on our analysis, we masked a skyline doublet that is close to the helium spectral feature; this doublet is located at 10,832.103 and 10,832.412 Å (*47*). For each spectrum, we first converted a wavelength range of 10,832.103 to 10,832.412 Å into its stellar rest frame (see below) and then used linear interpolation to approximate the fluxes.

Corrections to telluric absorption were not performed given we planned our observations strategically when telluric bands were widely separated from the helium spectral feature of HAT-P-32 Ab, as shifted by Earth’s barycentric motion (see Fig. 3).

Our reduced spectra are all in vacuum wavelengths. All spectra have S/Ns above 45 per pixel, i.e., >75 per resolution element, near the helium triplet at 10,833 Å; the median S/N is 85 per pixel. During the observation, the stellar companion B is outside the HPF target fiber, the radius of which (0.85′′) is only 29% of the angular separation between A and B components (2.94′′) (*44*, *45*). In addition, the B-to-A flux ratio is about 0.017 near the helium triplet at 10,833 Å [see figure 4 of (*41*)]; this ratio is comparable to the noise-to-signal ratio of A’s observed fluxes, with a median of 0.012 (=1/85). Thus, the stellar companion would have comparable fluxes with A’s flux uncertainties, even if both stellar components were observed by the same fiber. Therefore, the stellar companion HAT-P-32 B has negligible contamination to our observations of HAT-P-32 A + b.

### HPF relative RVs of HAT-P-32 A

We measured the relative RVs of HAT-P-32 A from the HET/HPF spectra following (*48*). We first applied the barycentric correction to all spectra using Astropy (*49*, *50*) and then multiplied each spectrum by a third-order polynomial, with coefficients determined in the steps described below, to match the fluxes of the science spectrum (observed on 19 September 2020 UT) with the highest S/N among our entire dataset. To create a master template, we carried out a cubic basic (B-spline) regression to data points from all these scaled spectra via the least-squares minimization implemented with a κ-sigma clipping that removes significant outliers from the residuals. We then jointly fitted the relative RV and the third-order polynomial coefficients for each spectral order of each spectrum by minimizing the χ^2^ between the given spectrum and the master template, over a grid of velocities (from −5 to +5 km s^−1^ with steps of 50 m s^−1^) following (*51*). During this process, we also masked the telluric absorption and sky emission features. We determined the RV value and variance of a given spectral order based on the χ^2^-velocity parabola and computed the final relative RV for each spectrum as the weighted mean and SE of RVs among all spectral orders. The spectrum with the highest S/N in our dataset thus provides the baseline for the measured relative RVs. Our resulting HPF relative RVs has typical uncertainties of 131 m s^−1^.

Given that HAT-P-32 A has an absolute RV of −23.21 ± 0.26 km s^−1^ based on (*19*), we thus added this value to the relative RV of each spectrum to obtain an absolute RV, with uncertainties added in quadrature. The computed absolute RVs of our data have a typical uncertainty below 0.3 km s^−1^, corresponding to a wavelength shift of only <0.01 Å near the helium triplet. We used the computed absolute RV of each spectrum to shift it into the stellar rest frame.

### EW measurements of helium and the calcium infrared triplets

We measured the EWs of the helium triplet in the stellar rest frame by integrating the line flux over 10,831.5 to 10,834.5 Å, with the pseudo-continuum approximated by a linear fit of the fluxes from two surrounding wavelength regions of 10,824 to 10,826 Å and 10,840 to 10,841 Å. The flux uncertainties are propagated into our resulting EWs in an analytical fashion. We also measured the EWs of the calcium infrared triplet (Ca ii IRT) at 8500, 8544, and 8644 Å, which probe the stellar chromospheric activity. These lines are thus used to validate that our observed significant excess helium absorption has a planetary rather than stellar origin [e.g., (*21*, *52*)]. To compute the EW of each triplet component, we defined the line wavelength as 8498 to 8503 Å with the pseudo-continuum established from 8491 to 8495 Å and 8505 to 8509 Å for Ca ii λλ8500; line wavelength as 8540 to 8549 Å with the pseudo-continuum from 8536 to 8537.5 Å, 8552 to 8556 Å, and 8562 to 8570 Å for Ca ii λλ8545; and line wavelength as 8661 to 8668 Å with the pseudo-continuum from 8658 to 8660 Å, 8672 to 8676 Å, and 8680 to 8688 Å for Ca ii λλ8665. For a given spectrum, we adopted the EW of the Ca ii IRT as the sum of all three components’ EWs. As shown in fig. S1, our measured Ca IRT EWs of spectra in pre, transit, and post subsets, when the escaping helium atmosphere and/or the planet are in transit, are all consistent with those of out-of-transit data in start and end subsets. Therefore, the observed helium excess spectral features likely originate from the planet’s upper atmosphere. This conclusion is supported by the observed low activity level from the ground-based optical light curves [e.g., (*53*)] and was also drawn by Czesla *et al.* (*21*) based on CARMENES data.

### Construction of the reference, transmission, and residual spectra

We resampled each spectrum in the stellar rest frame to the same wavelength grid of the spectrum that has the highest S/N at 10,833 Å. Then, we normalized the spectrum in the neighborhood of the helium triplet, with the pseudo-continuum established from a linear fit of fluxes from 10,824 to 10,826 Å and 10,840 to 10,841 Å as used in our EW measurements. For data collected from each of the three transit events and also from the long-term stellar activity monitoring, we derived a reference spectrum by computing the average of all spectra corresponding to orbital phases below −0.15 (i.e., start) or above +0.08 (i.e., end). We propagated the flux uncertainties in an analytical fashion and obtained a total of four reference spectra. As shown in fig. S2, our computed reference spectra all have consistent shapes and values in the neighboring wavelengths of the helium triplet, with different fluxes from the telluric absorption lines that are clearly separated from the helium feature. We then divided each normalized pre, transit, and post spectrum by its corresponding reference spectrum to produce transmission and residual (transmission 1) spectra (figs. S3 and S4). A telluric band, spanning 10,835 to 10,840 Å in the stellar rest frame, is near but separated from the helium feature in our residual spectra. Thus, our analysis cannot detect if there is any helium excess from a clump of the planet’s escaping atmosphere with an RV toward the host star with values of 48 to 187 km s^−1^; such signal is not predicted by our subsequent 3D hydrodynamic simulation for our target (see below).

### Gaussian models of the excess helium absorption

The observed wavelengths of helium excess reflect the RVs of helium upper atmosphere escaping HAT-P-32 b. To identify these wavelengths, we fitted a Gaussian profile $G(\lambda )=-A_{G}\;\exp\;[-{\left(\lambda -\mu_{G}\right)}^{2}/2\sigma_{G}^{2}]$ to each residual spectrum over 10,825 to 10,840 Å, with *A*_G_, μ_G_, and σ_G_ describing the transit depth, central wavelength, and SD wavelength of each excess absorption feature. We excluded telluric bands redward of the helium triplet, as well as wavelengths of sky emission lines whose fluxes were replaced by linear interpolation during the data processing. Some residual spectra observed near the end of the planet’s optical transit show broad absorption features that have slightly shorter wavelengths and shallower depths than the helium excess (e.g., the residual spectrum of the second transit event with the orbital phase near 0.012; fig. S3). These features line up with the weakest component of the helium triplet (Fig. 3) but contain an absorption component that is even more blueshifted [also see (*21*)]. We chose to exclude wavelengths of such feature in our fitting process, given that the goal of fitting Gaussian profiles is to identify wavelengths of the primary component of the excess helium absorption.

On the basis of our fitted *A*_G_ values, the depth of helium excess from each residual spectrum in the pre (6 spectra in total), transit (18 spectra in total), and post (7 spectra in total) subsets spans 2 to 3%, 4 to 7%, and 2 to 4%, respectively. The transit depth in each subset is further increased after stacking all residual spectra (Fig. 3). In addition, our observed helium excess has full widths at half maximum of 1.5 to 3 Å (i.e., 2.355σ_G_), comparable with the detected helium excess in other planetary systems [e.g., (*11*, *12*)]. To compute the RV of the escaping helium atmosphere of HAT-P-32 b, we further compared the fitted central wavelength μ_G_ of each residual spectrum with the mean rest wavelength of the strongest two components of the helium triplet at 10,833.26 Å. Our fitted μ_G_, σ_G_, and computed escaping RVs are presented in Fig. 2.

### Planetary orbital RV in the stellar rest frame

We computed the planet’s RV in the stellar rest frame as a function of the planet’s orbital phase (ϕ) based on the following equation

$${\mathrm{RV}}_{p}(\varphi )=-\left(1+\frac{1}{M_{p}/M_{*}}\right)\times K_{*}\;\{\cos\;[v(\varphi )+\omega_{*}]+e\;\cos\;\omega_{*}\}$$ (1)

where *M*_p_/$M_{*}$ is the planet-to-star mass ratio, $K_{*}$ is the semiamplitude of the host star’s RV, $\omega_{*}$ is the argument of the periastron of the host star’s orbit induced by the exoplanet, and *e* is the planet’s orbital eccentricity. The true anomaly, *v*, is converted from a given orbital phase based on the planet’s orbital period (*P*) and eccentricity (*e*), time of periastron (*T*_P_), and the time of the planet’s optical transit (*T*_C_).

We computed RV_p_(ϕ) for a circular planet orbit and adopted *M*_p_/$M_{*}$ = 4.81 × 10^4^ (*21*), $K_{*}$ = 83.4 m s^−1^ (*21*), *P* = 2.1500082 days (*20*), and *T*_C_ = 2,456,237.031 BJD_TDB_ (*20*). We simply assumed $\omega_{*}0^{\circ }$ and *T*_P_ = *T*_C_ − *P*/4 for using Eq. 1. We did not incorporate the uncertainties of orbital parameters in our calculation, given our analysis compares the planet’s RV with measured RVs of the escaping helium atmosphere under a qualitative perspective. Our computed RV_p_(ϕ) is presented in Fig. 2.

### Stacked residual spectra and the light curve of excess helium absorption

We shifted all residual spectra to an “escaping atmospheric rest frame” based on the computed RVs of the helium upper atmosphere escaping HAT-P-32 b, such that the helium excess feature in each shifted residual spectrum all lines up with the rest wavelength of the strongest component of the helium triplet at 10,833.26 Å. We then computed the stacked residual spectrum based on the weighted flux mean and uncertainty of all data in each of the pre, transit, and post subsets (Fig. 3). In addition to the excess helium absorption features, the stacked pre residual spectrum shows a deep absorption feature near 10,835 Å, which is caused by the telluric absorption mainly contributed by the pre residual spectrum with an orbital phase of −0.098 and −0.111 (fig. S4). The wavelength of this feature is outside the wavelength range used to compute the helium EWs (Fig. 1). To compute the light curve of excess helium absorption (Fig. 1), we integrated each residual spectrum in the escaping atmospheric rest frame over a window centered at 10,833.26 Å (the mean rest wavelength of the strongest two components of the helium triplet) with a width of ±1.8 Å (corresponding to an RV range of ±50 km s^−1^), with spectral uncertainties propagated in an analytical fashion.

### 3D hydrodynamic models

Our 3D hydrodynamic models made use of the Athena++ (magneto)hydrodynamics code (*54*), which uses an Eulerian algorithm with active and static mesh refinement capabilities. Our models simulated the properties of the interaction between the planetary outflow (with properties based on parameterized assumptions) and the stellar wind and the planet-star gravitational field [e.g., (*31*)]. We adopted a nearly isothermal ideal-gas equation of state with an adiabatic index γ = 1.0001 (allowing each of the stellar and planetary outflows to be nearly isothermal but with very different temperatures) and ignored any possible effects of stellar or planetary magnetic and radiation fields.

We solved the hydrodynamics equations in a rotating reference frame centered on the host star HAT-P-32 A. Our frame rotates with the planetary mean motion to minimize the orbital advection of planetary outflow across the coordinate mesh. We used a spherical-polar mesh that covers the full 4π of solid angle and extends in radius from HAT-P-32 A’s stellar radius of $R_{*}$ ≈ 9.5 × 10^10^ cm [or 1.37 ± 0.03 *R*_⊙_ as determined by (*20*)], to about 100 × $R_{*}$ of 9 × 10^12^ cm. With a semimajor axis of 0.034 au ~5 × 10^11^ cm (*19*, *20*, *41*) or 5.3 $R_{*}$, the orbit of HAT-P-32 b is covered by this coordinate mesh. Our base mesh uses 144 × 96 × 192 zones in the *r*, θ, ∅ coordinate, with the planet placed at θ = π/2. To increase the resolution of simulations near the planet’s orbit, we add static mesh refinement. Specifically, we refined an equatorial torus extending 2 × 10^11^ to 8 × 10^11^ cm in *r*, π/4 − 3π/4 in θ, and 0 − 2π in ∅ by one level above the base mesh or a factor of two higher spatial resolution. We also refined a box surrounding the planet location on the mesh by four levels of refinement (i.e., 16× higher spatial resolution than the base mesh) to capture the planet-scale outflow with sufficient resolution in our simulation.

We set our model parameters based on orbital properties (with *e* = 0) that have been derived for HAT-P-32 b (*19*, *21*). To model the planetary outflow, we used the hydrodynamic escape parameter

$$\lambda =\frac{GM_{p}}{R_{p}c_{s}^{2}}$$ (2)

where *M*_p_ is the planet’s mass, *R*_p_ is the planet radius, and *c*_s_ is the sound speed of the outflow. We adopted λ = 8 for the planetary wind in our models, implying an outflow temperature of ≈5750 K. This is a free parameter of our current models, and we find that λ particularly affects the stream kinematics, as well as the inferred planetary mass loss rate (*55*). To model the much hotter (≈10^6^ K), fast-expanding stellar wind, we adopted λ = 15 and replaced *M*_p_ and *R*_p_ to be the mass and radius of the host star, respectively, in the Eq. 2. The planetary mass loss rate in our model is 1.07 × 10^12^ g s^−1^ (≈1.7 × 10^-^^14^ *M*_⊙_ year^−1^). This implies a loss time scale for the planetary envelope of $M_{p}/{\dot{M}}_{p}\approx 3.8\times 10^{10}$ year. The stellar mass loss rate in our model, ≈7 × 10^-^^14^ *M*_⊙_ year^−1^, is on the order of typical main sequence mass loss rates (*56*).

We postprocessed these snapshots by iterating to find the stellar and planetary outflow ionization states in the stellar radiation field. To construct the spectral energy distribution of the host star, we combined the published XUV spectrum of τ Boo (0 to 1200 Å) (*57*), which has the same spectral type as HAT-P-32 A, and the BT-Settl model spectrum (>1200 Å) with an effective temperature of 6300 K and logarithmic surface gravity of log(*g*) = 4.5 dex; we further scaled this spectrum based on the XUV flux estimates from (*21*). We then cast rays from the star through the domain to an observer. The full methodology is presented in (*31*).

Figure 4 demonstrates a slice of gas density through the simulation model domain centered on HAT-P-32 A, showing that an outflow from HAT-P-32 b extends nearly along the orbital path both leading and trailing the planet. This outflow is shaped by its interactions with the much-faster stellar wind (as is visible from unstable interfaces between the higher-density planetary outflow and the lower-density stellar wind). However, the primary force shaping the planetary outflow is the tidal gravity of the star-planet system, which is particularly strong for HAT-P-32 b because the planet is nearly filling its Roche lobe (*21*). As a result, the planetary outflow is slow-moving by comparison to the planet’s orbital speed. The differential orbital frequency as a function of the distance from the star means that gas in the planetary outflow is advected into leading and trailing arms of the planet’s upper atmosphere.

**Fig. 7.**
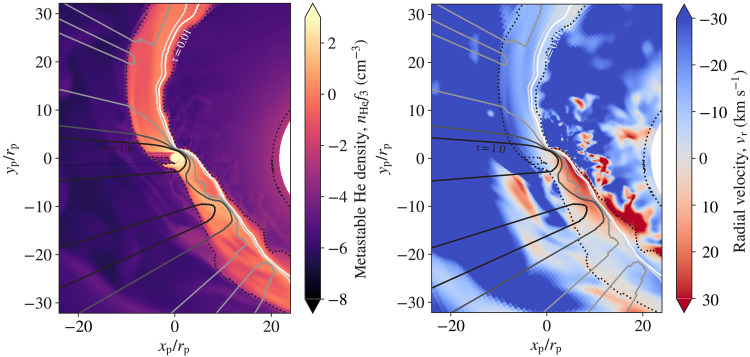
Number density and RV of the metastable helium in 3D simulations. The left panel shows the showing number density of metastable helium (left) and and the right panel shows the RV in a postprocessed simulation snapshot (same as Fig. 4) . Contours show the cumulative radial optical depths of 0.01 to 1 in half-dex steps (light to dark). The dotted contour shows the surface of *n*_He_*f*_3_10^−3^ cm^−3^. The extended tidal tails of mass loss in this HAT-P-32 Ab analog system imply significant optical depth of the planetary mass loss even far from the planet itself, in significant contrast to a more-spherical pattern of mass loss. Further, these tidal tails orbit the host star with little RV (or line-of-sight velocity for an observer) implying that their absorption signatures lie close to the stellar rest frame, which is in excellent agreement with observations (see Fig. 2).

The consequences for the observable properties of excess helium absorption of this outflow geometry are marked. Figure 7 presents the number density of metastable helium and the RV of gas in the region surrounding the planet. Overlying contours show the cumulative, radial optical depth from the host star in the metastable helium line. These contours show that the columnar structure of planetary outflow maintains relatively high optical depth even at large distances from the planet. This can be contrasted to a spherical outflow, in which the optical depth drops steeply with distance above the planetary surface. For the observable properties of HAT-P-32 b’s transit, this means that the extended absorption in pre and post phases (e.g., Fig. 1) can be attributed to columns of planetary outflow that are strongly shaped by orbital advection into leading and trailing tails.

Figure 7 also shows the RV of material with the same overlying contours of optical depth. We see that on the leading arm, planetary material has a slight redshift, implying flow toward the host star, while material in the trailing arm is slightly blueshifted. The RVs of gas are low compared to the planet’s orbital velocity (~170 km s^−1^). The leading and trailing tails of the planet’s escaping atmosphere, therefore, lie close to the stellar rest frame as most of its motion is along the orbit, perpendicular to an observer during transit. Several properties of the model affect the kinematics of the tidal tails of planetary mass loss. We find that one of the strongest effects is the characteristic temperature of the planetary outflow (controlled by the parameter λ in our models). Cooler tidal tails are shaped more strongly by the tidal potential and are broadened less by their own thermal expansion. This suggests that more sophisticated modeling that fully reproduces the EW light curve (Fig. 1) and kinematics as a function of phase (Fig. 2) will be very constraining about the planetary outflow’s thermodynamics.

The pre and post tails of material need not share an identical temperature (as they currently do in our hydrodynamic models). We find that cooler post tails better reproduce the redshifts observed several hours after mid-transit. In addition, it is important to highlight that there are physical processes not included in our models that could influence tidal tail kinematics. We have not included momentum deposition by the stellar radiation field on the outflow directly in our models, nor have we systematically varied the orbital eccentricity, the relative strength of the stellar wind, or the thermodynamics of the planetary outflow. Stellar wind and radiation pressure act similarly, applying a roughly *r*^−2^-scaled radial pressure away from the star. Increasing these effects blueshifts the trailing tail and can erode the leading tail until the outflow forms a cometary morphology (*31*). Eccentricity introduces waves into the tails, perhaps including subtle RV signatures. Because these effects interact nonlinearly, sophisticated hydrodynamic simulations are needed to explore this phase space and to determine to what degree the observational constraints uniquely determine HAT-P-32 A + b’s properties.

We also supplemented our 3D modeling with 1D hydrodynamic models [following (*58*, *59*)] of planetary outflows of varying temperatures, with otherwise equivalent assumptions. At identical temperatures and EW, these 1D models predict ~2× higher planetary mass loss rate because they neglect the compression of material into tidal tails. The 1D models also suggest that the best-fitting mass-loss rate scales as ${\dot{M}}_{p}\propto T$ (i.e., ${\dot{M}}_{p}\;\propto_{p}^{1}\;\lambda_{p}^{-1}$, where *T* is the outflow temperature), thus giving an indication of how varying the uncertain planetary outflow thermodynamics affects the inferred planetary mass loss rate [also see (*55*)].

## Supplementary Materials

### This PDF file includes:

Figs. S1 to S4 Table S1 Legend for data S1 References
