# Supplementary material for: Giant tidal tails of helium escaping the hot Jupiter HAT-P-32 b
Source: Sci Adv. 2023 Jun 7;9(23):eadf8736. doi: 10.1126/sciadv.adf8736 (PMC10246913; doi:10.1126/sciadv.adf8736)
Supplement: Supplementary file 1 — Figs. S1 to S4 Table S1 Legend for data S1 References [file sciadv.adf8736_sm.pdf]

Science Advances
AAAS

Supplementary Materials for

Giant tidal tails of helium escaping the hot Jupiter HAT-P-32 b

Zhoujian Zhang *et al.*

Corresponding author: Zhoujian Zhang, zhangdirac@gmail.com

Sci. Adv. **9**, eadf8736 (2023)
DOI: 10.1126/sciadv.adf8736

The PDF file includes:

Figs. S1 to S4
Table S1
Legend for data S1
References

Other Supplementary Material for this manuscript includes the following:

Data S1

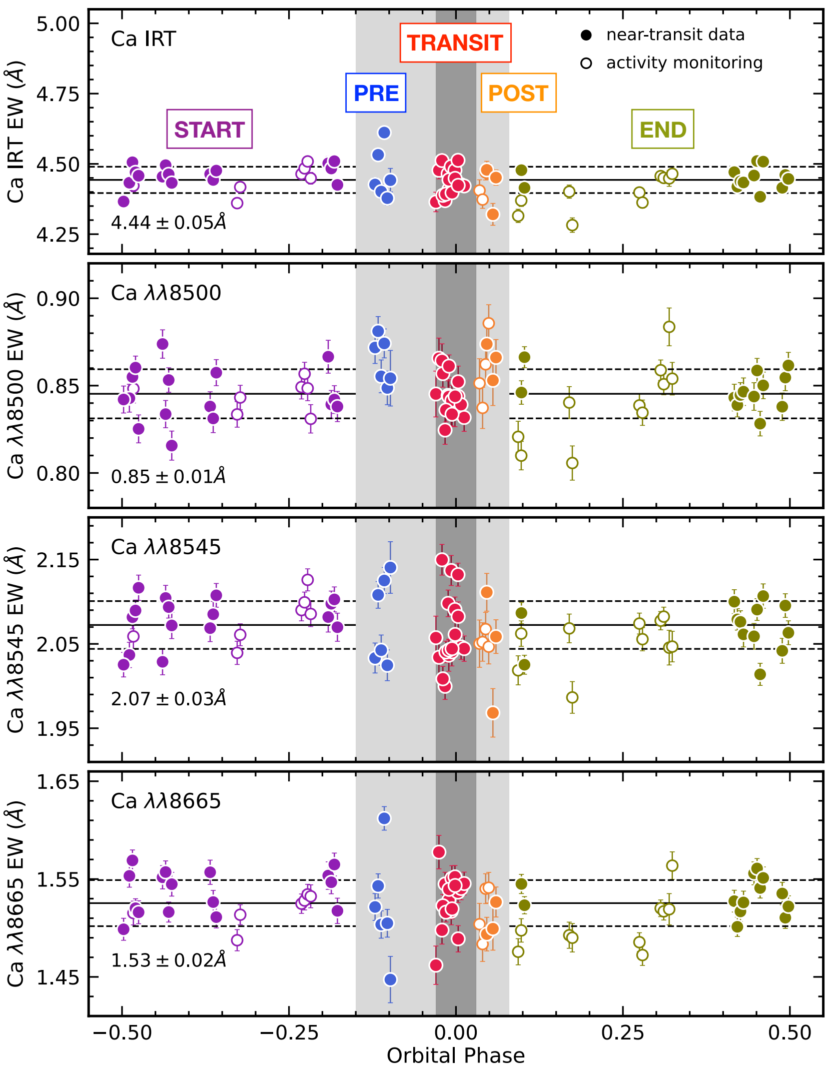

Fig. S1. Measured equivalent widths (EWs) of the calcium infrared triplet are not correlated with the planet's orbital phase, suggesting the observed excess helium absorption originates from the planet's exospheres rather than stellar activity indicators. The top panel presents the total EWs of the Ca triplet, computed as the sum of individual components' EWs as shown in the remaining three panels. The format is the same as Fig. 1, with the weighted mean and root-mean-square of EWs during the START and END phases labeled at the bottom left of each panel.

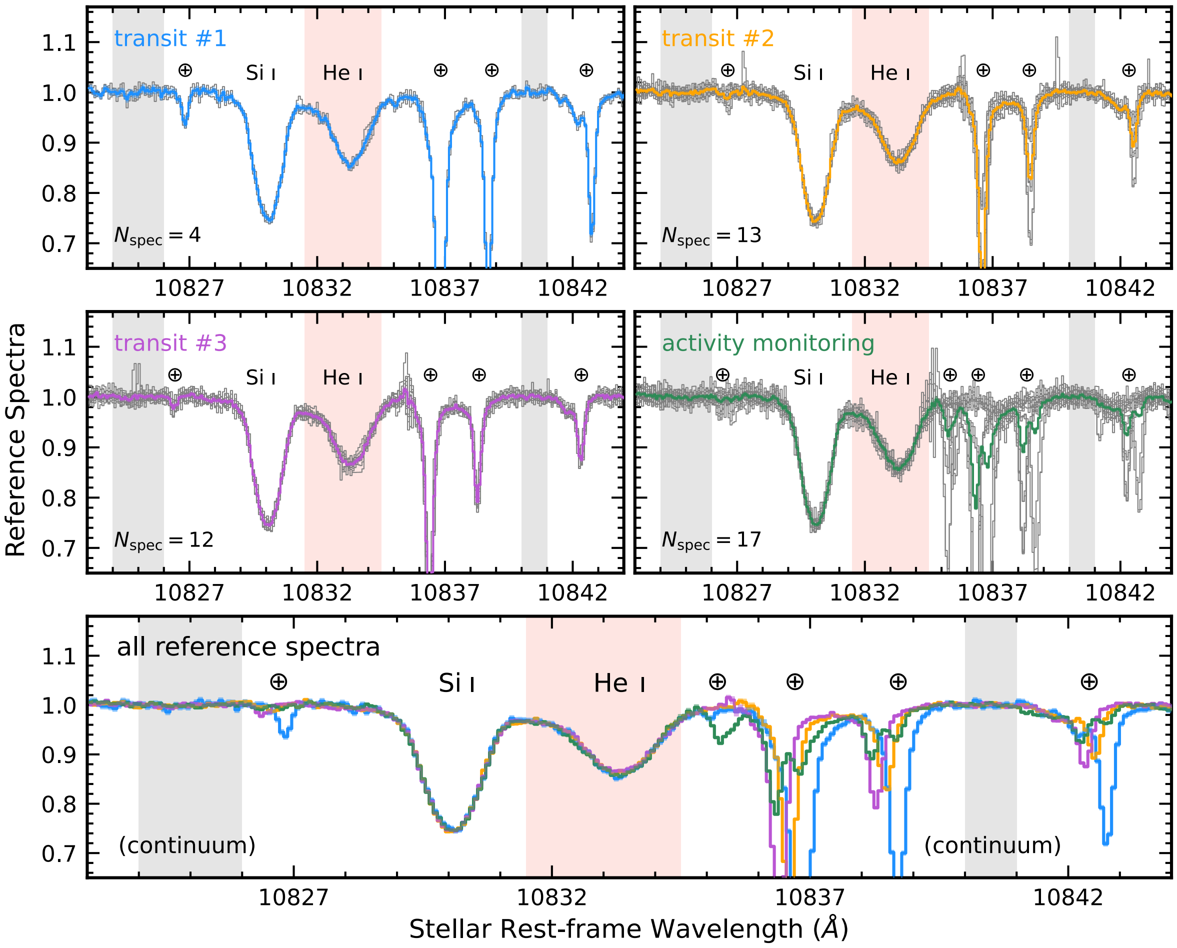

Fig. S2. Reference spectra for the first (top left; blue), second (top right; orange), and third (middle left; purple) transit event, as well as the long-term stellar activity monitoring (middle right; green). In each panel, we label the number of spectra used for computing the reference spectra (N_{spec}) and overlay individual spectra as gray lines. All four reference spectra have consistent shapes and fluxes near the helium and silicon features (bottom panel), with different strengths of telluric absorption (labeled by "$\oplus$") that are widely separated from the helium triplet at 10833 Å. In each panel, the red and gray shades show the wavelength ranges used for computing the line flux and the pseudo-continuum of the helium EW, respectively.

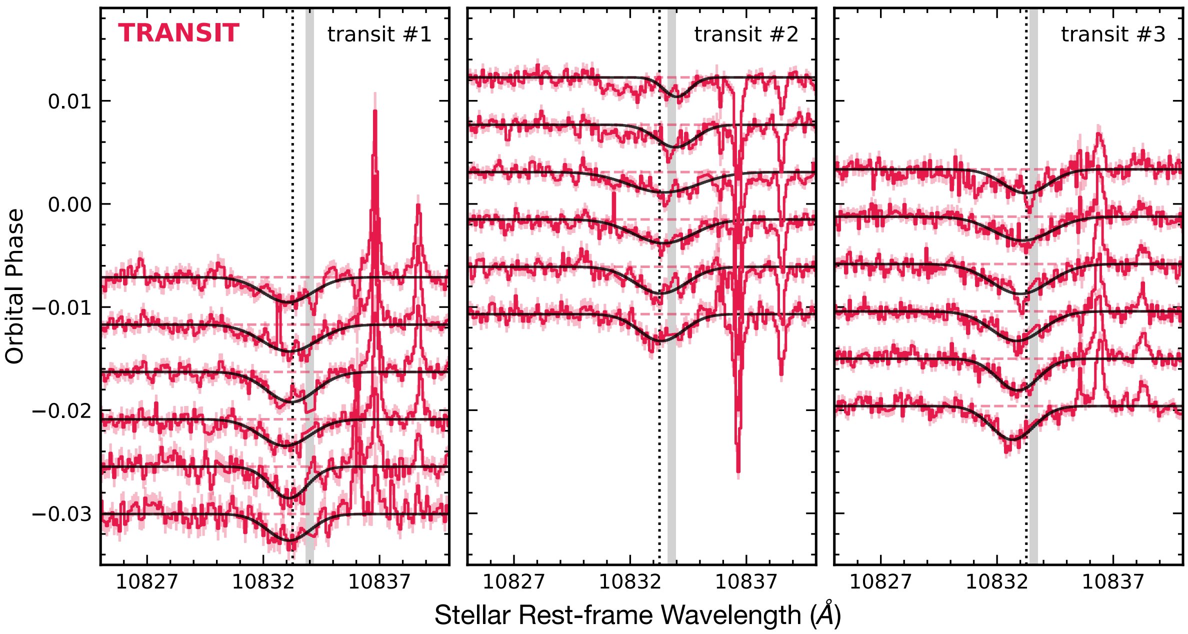

Fig. S3. Residual spectra (solid lines; scaled by a factor of 0.05) and 1σ uncertainties (shades) in the TRANSIT subset of all three transit events ordered by the orbital phase, showing significant excess helium absorption features. We marked the rest wavelength of the two strongest and blended components of the helium triplet at 10833.26 Å (dotted vertical line). Vertical gray shades highlight the wavelength ranges of the OH skyline doublet, over which we masked and approximated the spectral fluxes based on linear interpolation. A Gaussian fit to the Helium excess in each residual spectrum is overlaid (black). Sharp spikes and absorption features near 10837 Å are due to the telluric absorption features that are widely separated from the helium signal.

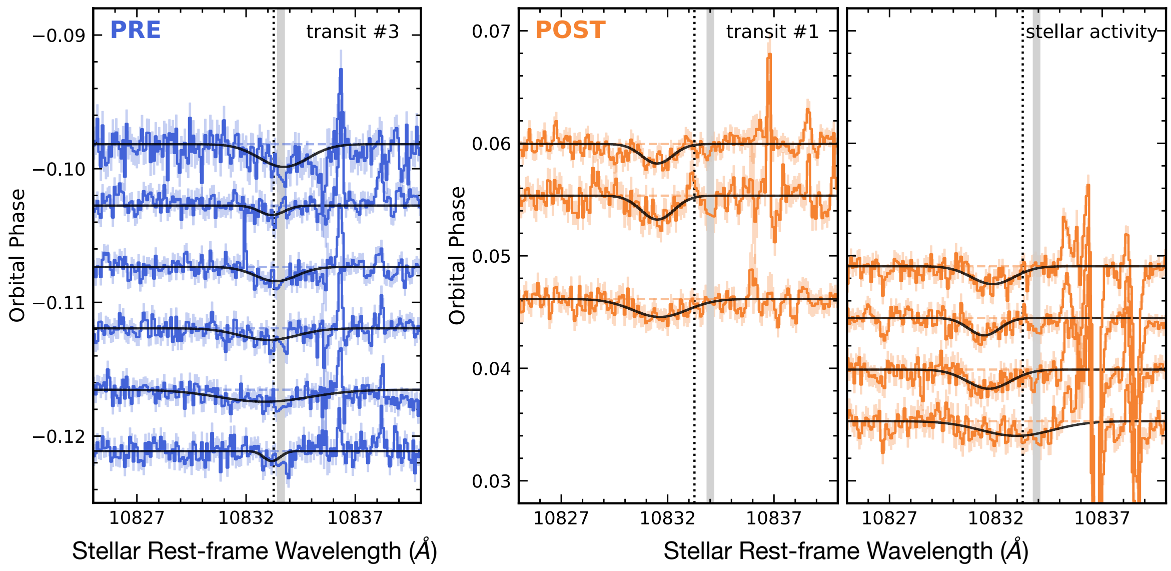

Fig. S4. Residual spectra (solid lines; scaled by a factor of 0.05) and 1σ uncertainties (shades) in the PRE (left; orbital phase in $[-0.15, -0.03]$) and POST (right; orbital phase in $[+0.03, +0.08]$) subsets ordered by the orbital phase. The PRE residual spectra are only from the third transit event, and the POST residual spectra are from both the first transit event and our long-term stellar variability monitoring. The format is the same as Fig. S3.

System	$\delta R_p/H_{\rm eq}$	$R_p/R_{\rm RL}$	**Planet surface gravity** (g cm^{-2})	**Planet** $T_{\rm eq}$ (K)	**Incident XUV Flux** (W m^{-2})	**Host Star** $T_{\rm eff}$ **(K)**	**Reference**
K2-136 c	–	–	–	425 ± 21	0.960 ± 0.050	4499 ± 50	(*62–65*)
K2-100 b	–	0.294 ± 0.035	1419 ± 418	1841 ± 41	35.700 ± 6.400	5945 ± 110	(*66–67*)
V1298 Tau c	–	–	–	968 ± 31	329.100 ± 11.550	4970 ± 120	(*68–70*)
TRAPPIST-1 b	–	0.023 ± 0.001	1080 ± 55	400 ± 7	3.000 ± 0.400	2559 ± 50	(*71–73*)
TRAPPIST-1 e	–	0.093 ± 0.009	801 ± 33	251 ± 4	0.400 ± 0.070	2559 ± 50	(*71–74*)
TRAPPIST-1 f	–	0.070 ± 0.002	932 ± 35	219 ± 4	0.270 ± 0.040	2559 ± 50	(*71–74*)
WASP-177 b	–	0.483 ± 0.155	494 ± 298	1142 ± 32	3.500 ± 4.650	5017 ± 70	(*75–76*)
GJ 9827 d	< 15	0.116 ± 0.009	966 ± 200	680 ± 25	2.450 ± 3.265	4340 ± 46	(*33, 77–78*)
V1298 Tau b	–	–	–	677 ± 22	78.600 ± 2.750	4970 ± 120	(*68–70*)
HD 97658 b	< 89	0.075 ± 0.004	1803 ± 227	751 ± 12	1.110 ± 1.480	5212 ± 43	(*77,79*)
55 Cnc e	< 12	0.356 ± 0.008	2223 ± 111	1958 ± 15	7.400 ± 9.850	5712 ± 18	(*80–82*)
KELT-9 b	< 40	0.527 ± 0.059	1989 ± 589	4050 ± 180	< 0.150	10170 ± 450	(*12, 83*)
GJ 9827 b	< 84	0.253 ± 0.010	1932 ± 200	1172 ± 43	37.000 ± 49.500	4340 ± 46	(*33, 77–78*)
GJ 436 b	< 38	0.238 ± 0.018	1252 ± 160	649 ± 60	0.197 ± 0.007	3350 ± 300	(*12, 84–85*)
WASP-80 b	< 34	0.298 ± 0.017	1336 ± 121	825 ± 19	6.281 ± 4.711	4143 ± 93	(*86–87*)
WASP-127 b	< 16	0.482 ± 0.024	238 ± 29	1400 ± 24	0.058 ± 0.034	5620 ± 85	(*88–90*)
WASP-76 b	< 36	0.649 ± 0.024	678 ± 42	2160 ± 40	< 94.000	6250 ± 100	(*91–92*)
HAT-P-18 b	13 ± 2	0.280 ± 0.017	493 ± 60	852 ± 28	8.000 ± 10.500	4803 ± 80	(*39, 84, 93*)
TOI 1430 b	171 ± 61	0.089 ± 0.013	1550 ± 525	813	6.800 ± 9.050	5067 ± 60	(*36*)
V1298 Tau d	–	–	–	845 ± 27	191.000 ± 6.650	4970 ± 120	(*68–70*)
TOI 560 b	121 ± 40	0.116 ± 0.013	1278 ± 408	714 ± 21	5.100 ± 1.300	4511 ± 110	(*34, 94*)
TOI 1683 b	126 ± 51	0.171 ± 0.028	1463 ± 535	927	12.000 ± 16.000	4539 ± 100	(*36*)
HD 209458 b	49 ± 3	0.352 ± 0.012	936 ± 57	1449 ± 12	1.004 ± 0.284	6091 ± 10	(*12, 84, 95–97*)
TOI 2076 b	189 ± 9	0.101 ± 0.003	1371 ± 48	797 ± 12	9.500 ± 12.650	5200 ± 70	(*36, 98*)
HD 189733 b	59 ± 4	0.325 ± 0.015	2194 ± 159	1201 ± 12	16.750 ± 0.028	5052 ± 16	(*12, 84, 96, 99–100*
HAT-P-11 b	99 ± 14	0.171 ± 0.009	1127 ± 145	875 ± 15	2.109 ± 0.124	4780 ± 50	(*11, 101–102*)
GJ 3470 b	79 ± 18	0.265 ± 0.021	650 ± 86	593 ± 96	1.435 ± 0.008	3600 ± 100	(*61, 103–104*)
GJ 1214 b	51 ± 5	0.312 ± 0.011	1065 ± 70	596 ± 19	0.640 ± 0.855	3250 ± 100	(*77, 103–104*)
WASP-52 b	68 ± 6	0.578 ± 0.018	707 ± 45	1315 ± 35	24.800 ± 33.150	5000 ± 100	(*35, 76, 107*)
WASP-69 b	99 ± 14	0.367 ± 0.026	583 ± 73	963 ± 18	4.170 ± 0.566	4700 ± 50	(*12, 96, 108*)
WASP-107 b	96 ± 13	0.299 ± 0.017	345 ± 43	736 ± 17	2.664 ± 1.050	4425 ± 70	(*10, 12, 28, 109–112*)
HAT-P-32 b	119 ± 8	0.650 ± 0.019	453 ± 27	1835 ± 6	165.000 ± 83.000	6001 ± 88	(*19–21*), This work

Table S1. Properties of all planetary systems with detections, non-detections, or upper-limit constraints of excess helium absorption. The $\delta_{R_p}/H_{\rm eq}$ parameter expresses the equivalent height of the planets' helium upper atmospheres in units of their scale height at equilibrium temperature ($T_{\rm eq}$). The $R_P/R_{\rm RL}$ parameter expresses the Roche-lobe filling, computed as the ratio between the planetary radii and the radii of their Roche Lobes.

Data S1. Measured spectral and physical properties for individual HPF spectra of HAT-P-32 A+b. This file includes, for each HPF spectrum, the file name, observing date and the corresponding orbital phase of the planet, barycentric and stellar RV, equivalent widths of the helium 10833 Å feature and the calcium infrared triplet of HAT-P-32 A+b, central wavelength of the helium excess absorption feature of HAT-P-32 b, and the equivalent width of the planet's helium excess absorption.

REFERENCES AND NOTES

1. T. Mazeh, T. Holczer, S. Faigler, Dearth of short-period Neptunian exoplanets: A desert in period-mass and period-radius planes. *Astron. Astrophys.* **589**, 78–81 (2016).

2. J. E. Owen, Y. Wu, Kepler planets: A tale of evaporation. *Astrophys. J.* **775**, 105 (2013).

3. E. D. Lopez, J. J. Fortney, The role of core mass in controlling evaporation: The kepler radius distribution and the Kepler-36 density dichotomy. *Astrophys. J.* **776**, 11 (2013).

4. J. E. Owen, Y. Wu, The evaporation valley in the Kepler planets. *Astrophys. J.* **847**, 29 (2017).

5. S. Ginzburg, H. E. Schlichting, R. Sari, Core-powered mass-loss and the radius distribution of small exoplanets. *Mon. Notices Royal Astron. Soc.* **476**, 759–765 (2018).

6. A. Gupta, H. E. Schlichting, Sculpting the valley in the radius distribution of small exoplanets as a by-product of planet formation: The core-powered mass-loss mechanism. *Mon. Notices Royal Astron. Soc.* **487**, 24–33 (2019).

7. A. Gupta, H. E. Schlichting, Signatures of the core-powered mass-loss mechanism in the exoplanet population: Dependence on stellar properties and observational predictions. *Mon. Notices Royal Astron. Soc.* **493**, 792–806 (2020).

8. R. O. P. Loyd, E. L. Shkolnik, A. C. Schneider, T. Richey-Yowell, T. S. Barman, S. Peacock, I. Pagano, Current population statistics do not favor photoevaporation over core-powered mass loss as the dominant cause of the exoplanet radius gap. *Astrophys. J.* **890**, 23 (2020).

9. J. G. Rogers, A. Gupta, J. E. Owen, H. E. Schlichting, Photoevaporation versus core-powered mass-loss: Model comparison with the 3D radius gap. *Mon. Notices Royal Astron. Soc.* **508**, 5886–5902 (2021).

10. J. J. Spake, D. K. Sing, T. M. Evans, A. Oklopčić, V. Bourrier, L. Kreidberg, B. V. Rackham, J. Irwin, D. Ehrenreich, A. Wyttenbach, H. R. Wakeford, Y. Zhou, K. L. Chubb, N. Nikolov, J. M. Goyal, G. W. Henry, M. H. Williamson, S. Blumenthal, D. R. Anderson, C. Hellier, D. Charbonneau,

S. Udry, N. Madhusudhan, Helium in the eroding atmosphere of an exoplanet. *Nature* **557**, 68–70 (2018).

11. R. Allart, V. Bourrier, C. Lovis, D. Ehrenreich, J. J. Spake, A. Wyttenbach, L. Pino, F. Pepe, D. K. Sing, A. Lecavelier des Etangs, Spectrally resolved helium absorption from the extended atmosphere of a warm Neptune-mass exoplanet. *Science* **362**, 1384–1387 (2018).

12. L. Nortmann, E. Pallé, M. Salz, J. Sanz-Forcada, E. Nagel, F. J. Alonso-Floriano, S. Czesla, F. Yan, G. Chen, I. A. G. Snellen, M. Zechmeister, J. H. M. M. Schmitt, M. López-Puertas, N. Casasayas-Barris, F. F. Bauer, P. J. Amado, J. A. Caballero, S. Dreizler, T. Henning, M. Lampón, D. Montes, K. Molaverdikhani, A. Quirrenbach, A. Reiners, I. Ribas, A. Sánchez-López, P. C. Schneider, M. R. Zapatero Osorio, Ground-based detection of an extended helium atmosphere in the Saturn-mass exoplanet WASP-69b. *Science* **362**, 1388–1391 (2018).

13. S. Mahadevan, L. Ramsey, C. Bender, R. Terrien, J. T. Wright, S. Halverson, F. Hearty, M. Nelson, A. Burton, S. Redman, S. Osterman, S. Diddams, J. Kasting, M. Endl, R. Deshpande, The Habitable-zone Planet Finder: A stabilized fiber-fed NIR spectrograph for the Hobby-Eberly Telescope, in *Ground-Based and Airborne Instrumentation for Astronomy IV* (SPIE, 2012), vol. 8446, pp. 624–637.

14. S. Mahadevan, L. W. Ramsey, R. Terrien, S. Halverson, A. Roy, F. Hearty, E. Levi, G. K. Stefansson, P. Robertson, C. Bender, C. Schwab, M. Nelson, The Habitable-zone Planet Finder: A status update on the development of a stabilized fiber-fed near-infrared spectrograph for the for the Hobby-Eberly Telescope, in *Ground-Based and Airborne Instrumentation for Astronomy V* (SPIE, 2014), vol. 9147, pp. 543–552.

15. S. Mahadevan, T. Anderson, E. Balderrama, C. F. Bender, E. Bevins, S. Blakeslee, A. Cole, D. Conran, S. Diddams, A. Dykhouse, J. Darling, C. Fredrick, S. Halverson, F. Hearty, J. Jennings, K. Kaplan, S. Kanodia, E. Levi, E. Lubar, A. J. Metcalf, A. Monson, J. Ninan, C. Nitroy, L. Ramsey, P. Robertson, A. Roy, C. Schwab, M. Shetrone, R. Spencer, G. Stefansson, R. Terrien, J. Wright, The Habitable-zone Planet Finder: Engineering and commissioning on the Hobby Eberly telescope (conference presentation), in *Ground-based and Airborne Instrumentation for Astronomy VII* (SPIE, 2018), vol. 10702, p. 1070214.

16. L. W. Ramsey, M. T. Adams, T. G. Barnes, J. A. Booth, M. E. Cornell, J. R. Fowler, N. I. Gaffney, J. W. Glaspey, J. M. Good, G. J. Hill, P. W. Kelton, V. L. Krabbendam, L. Long, P. J. McQueen, F. B. Ray, R. L. Ricklefs, J. Sage, T. A. Sebring, W. J. Spiesman, M. Steiner, Early performance and present status of the Hobby-Eberly Telescope, in *Advanced Technology Optical/IR Telescopes VI* (SPIE, 1998), vol. 3352, p. 34–42.

17. M. Shetrone, M. E. Cornell, J. R. Fowler, N. Gaffney, B. Laws, J. Mader, C. Mason, S. Odewahn, B. Roman, S. Rostopchin, D. P. Schneider, J. Umbarger, A. Westfall, Ten year review of queue scheduling of the Hobby-Eberly Telescope. *Publ. Astron. Soc. Pac.* **119**, 556–566 (2007).

18. G. J. Hill, H. Lee, P. J. MacQueen, A. Kelz, N. Drory, B. L. Vattiat, J. M. Good, J. Ramsey, H. Kriel, T. Peterson, D. L. DePoy, K. Gebhardt, J. L. Marshall, S. E. Tuttle, S. M. Bauer, T. S. Chonis, M. H. Fabricius, C. Froning, M. Häuser, B. L. Indahl, T. Jahn, M. Landriau, R. Leck, F. Montesano, T. Prochaska, J. M. Snigula, G. Zeimann, R. Bryant, G. Damm, J. R. Fowler, S. Janowiecki, J. Martin, E. Mrozinski, S. Odewahn, S. Rostopchin, M. Shetrone, R. Spencer, E. M. Cooper, T. Armandroff, R. Bender, G. Dalton, U. Hopp, E. Komatsu, H. Nicklas, L. W. Ramsey, M. M. Roth, D. P. Schneider, C. Sneden, M. Steinmetz, The HETDEX instrumentation: Hobby-Eberly Telescope wide-field upgrade and VIRUS. *Astronom. J.* **162**, 298 (2021).

19. J. D. Hartman, G. Á. Bakos, G. Torres, D. W. Latham, G. Kovács, B. Béky, S. N. Quinn, T. Mazeh, A. Shporer, G. W. Marcy, A. W. Howard, D. A. Fischer, J. A. Johnson, G. A. Esquerdo, R. W. Noyes, D. D. Sasselov, R. P. Stefanik, J. M. Fernandez, T. Szklenár, J. Lázár, I. Papp, P. Sári, HAT-P-32b and HAT-P-33b: Two highly inflated hot Jupiters transiting high-jitter stars. *Astrophys. J.* **742**, 59–77(2011).

20. Y.-H. Wang, S. Wang, T. C. Hinse, Z.-Y. Wu, A. B. Davis, Y. Hori, J.-N. Yoon, W. Han, J.-D. Nie, H.-G. Liu, H. Zhang, J.-L. Zhou, R. A. Wittenmyer, X.-Y. Peng, G. Laughlin, Transiting exoplanet monitoring project (TEMP). V. Transit follow up for HAT-P-9b, HAT-P-32b, and HAT-P-36b. *Astronom. J.* **157**, 82 (2019).

21. S. Czesla, M. Lampón, J. Sanz-Forcada, A. García Muñoz, M. López-Puertas, L. Nortmann, D. Yan, E. Nagel, F. Yan, J. H. M. M. Schmitt, J. Aceituno, P. J. Amado, J. A. Caballero, N. Casasayas-Barris, T. Henning, S. Khalafinejad, K. Molaverdikhani, D. Montes, E. Pallé, A. Reiners, P. C.

Schneider, I. Ribas, A. Quirrenbach, M. R. Zapatero Osorio, M. Zechmeister, Hα and He I absorption in HAT-P-32 b observed with CARMENES - Detection of Roche lobe overflow and mass loss. *Astron. Astrophys.* **657**, 10.1051/0004-6361/202039919 (2021).

22. A. Quirrenbach, P. J. Amado, J. A. Caballero, R. Mundt, A. Reiners, I. Ribas, W. Seifert, M. Abril, J. Aceituno, F. J. Alonso-Floriano, M. Ammler-von Eiff, R. Antona Jiménez, H. Anwand-Heerwart, M. Azzaro, F. Bauer, D. Barrado, S. Becerril, V. J. S. Béjar, D. Benítez, Z. M. Berdiñas, M. C. Cárdenas, E. Casal, A. Claret, J. Colomé, M. Cortés-Contreras, S. Czesla, M. Doellinger, S. Dreizler, C. Feiz, M. Fernández, D. Galadí, M. C. Gálvez-Ortiz, A. García-Piquer, M. L. García-Vargas, R. Garrido, L. Gesa, V. Gómez Galera, E. González Álvarez, J. I. González Hernández, U. Grözinger, J. Guàrdia, E. W. Guenther, E. de Guindos, J. Gutiérrez-Soto, H. -J. Hagen, A. P. Hatzes, P. H. Hauschildt, J. Helmling, T. Henning, D. Hermann, L. Hernández Castaño, E. Herrero, D. Hidalgo, G. Holgado, A. Huber, K. F. Huber, S. Jeffers, V. Joergens, E. de Juan, M. Kehr, R. Klein, M. Kürster, A. Lamert, S. Lalitha, W. Laun, U. Lemke, R. Lenzen, M. López del Fresno, B. López Martí, J. López-Santiago, U. Mall, H. Mandel, E. L. Martín, S. Martín-Ruiz, H. Martínez-Rodríguez, C. J. Marvin, R. J. Mathar, E. Mirabet, D. Montes, R. Morales Muñoz, A. Moya, V. Naranjo, A. Ofir, R. Oreiro, E. Pallé, J. Panduro, V.-M. Passegger, A. Pérez-Calpena, D. Pérez Medialdea, M. Perger, M. Pluto, A. Ramón, R. Rebolo, P. Redondo, S. Reffert, S. Reinhardt, P. Rhode, H. -W. Rix, F. Rodler, E. Rodríguez, C. Rodríguez-López, E. Rodríguez-Pérez, R. -R. Rohloff, A. Rosich, E. Sánchez-Blanco, M. A. Sánchez Carrasco, J. Sanz-Forcada, L. F. Sarmiento, S. Schäfer, J. Schiller, C. Schmidt, J. H. M. M. Schmitt, E. Solano, O. Stahl, C. Storz, J. Stürmer, J. C. Suárez, R. G. Ulbrich, G. Veredas, K. Wagner, J. Winkler, M. R. Zapatero Osorio, M. Zechmeister, F. J. Abellán de Paco, G. Anglada-Escudé, C. del Burgo, A. Klutsch, J. L. Lizon, M. López-Morales, J. C. Morales, M. A. C. Perryman, S. M. Tulloch, W. Xu, CARMENES instrument overview, in *Ground-Based and Airborne Instrumentation for Astronomy V* (SPIE, 2014), vol. 9147, pp. 531–542.

23. J. P. Ninan, C. F. Bender, S. Mahadevan, E. B. Ford, A. J. Monson, K. F. Kaplan, R. C. Terrien, A. Roy, P. M. Robertson, S. Kanodia, G. K. Stefansson, The Habitable-zone Planet Finder: Improved flux image generation algorithms for H2RG up-the-ramp data, in *High Energy, Optical, and Infrared Detectors for Astronomy VIII* (SPIE, 2018), vol. 10709, pp. 694–704.

24. K. F. Kaplan, C. F. Bender, R. C. Terrien, J. Ninan, A. Roy, S. Mahadevan, The algorithms behind the HPF and NEID pipeline. *ASPCS* **523**, 567–570 (2019).

25. A. J. Metcalf, T. Anderson, C. F. Bender, S. Blakeslee, W. Brand, D. R. Carlson, W. D. Cochran, S. A. Diddams, M. Endl, C. Fredrick, S. Halverson, D. D. Hickstein, F. Hearty, J. Jennings, S. Kanodia, K. F. Kaplan, E. Levi, E. Lubar, S. Mahadevan, A. Monson, J. P. Ninan, C. Nitroy, S. Osterman, S. B. Papp, F. Quinlan, L. Ramsey, P. Robertson, A. Roy, C. Schwab, S. Sigurdsson, K. Srinivasan, G. Stefansson, D. A. Sterner, R. Terrien, A. Wolszczan, J. T. Wright, G. Ycas, Stellar spectroscopy in the near-infrared with a laser frequency comb. *Optica* **6**, 233–239 (2019).

26. M. Gully-Santiago, J. Luna, C. Morley, K. Kaplan, A. Ganesh, E. Sawczynec, J. Burke, D. Krolikowski, Astronomical échelle spectroscopy data analysis with muler. *J. Open Source Softw.* **7**, 4302 (2022).

27. J. E. Owen, F. C. Adams, Magnetically controlled mass-loss from extrasolar planets in close orbits. *Mon. Notices Royal Astron. Soc.* **444**, 3761–3779 (2014).

28. J. J. Spake, A. Oklopčić, L. A. Hillenbrand, The posttransit tail of WASP-107b observed at 10830 Å. *Astron. J.* **162**, 284–292 (2021).

29. D. Ehrenreich, V. Bourrier, P. J. Wheatley, A. L. des Etangs, G. Hébrard, S. Udry, X. Bonfils, X. Delfosse, J. M. Désert, D. K. Sing, A. Vidal-Madjar, A giant comet-like cloud of hydrogen escaping the warm Neptune-mass exoplanet GJ 436b. *Nature* **522**, 459–461 (2015).

30. B. Lavie, D. Ehrenreich, V. Bourrier, A. Lecavelier des Etangs, A. Vidal-Madjar, X. Delfosse, A. Gracia Berna, K. Heng, N. Thomas, S. Udry, P. J. Wheatley, The long egress of GJ 436b's giant exosphere. *Astron. Astrophys.* **605**, L7 (2017).

31. M. MacLeod, A. Oklopčić, Stellar wind confinement of evaporating exoplanet atmospheres and its signatures in 1083 nm observations. *Astrophys. J.* **926**, 226–238 (2022).

32. A. Oklopčić, Helium absorption at 1083 nm from extended exoplanet atmospheres: Dependence on stellar radiation. *Astrophys. J.* **881**, 133–140 (2019).

33. I. Carleo, A. Youngblood, S. Redfield, N. C. Barris, T. R. Ayres, H. Vannier, L. Fossati, E. Palle, J. H. Livingston, A. F. Lanza, P. Niraula, J. D. Alvarado-Gómez, G. Chen, D. Gandolfi, E. W. Guenther, J. L. Linsky, E. Nagel, N. Narita, L. Nortmann, E. L. Shkolnik, M. Stangret, A multiwavelength look at the GJ 9827 system: No evidence of extended atmospheres in GJ 9827b and d from HST and CARMENES data. *Astron. J.* **161**, 136–147 (2021).

34. M. Zhang, H. A. Knutson, L. Wang, F. Dai, O. Barragán, Escaping helium from TOI 560.01, a young mini-Neptune. *Astron. J.* **163**, 67–81 (2022).

35. G. Hébrard, A. Collier Cameron, D. J. A. Brown, R. F. Díaz, F. Faedi, B. Smalley, D. R. Anderson, D. Armstrong, S. C. C. Barros, J. Bento, F. Bouchy, A. P. Doyle, B. Enoch, Y. Gómez Maqueo Chew, E. M. Hébrard, C. Hellier, M. Lendl, T. A. Lister, P. F. L. Maxted, J. McCormac, C. Moutou, D. Pollacco, D. Queloz, A. Santerne, I. Skillen, J. Southworth, J. Tregloan-Reed, A. H. M. J. Triaud, S. Udry, M. Vanhuysse, C. A. Watson, R. G. West, P. J. Wheatley, WASP-52b, WASP-58b, WASP-59b, and WASP-60b: Four new transiting close-in giant planets. *Astron. Astrophys.* **549**, 1–11 (2013).

36. P. P. Eggleton, Approximations to the radii of Roche lobes. *Astrophys. J.* **268**, 368–369 (1983).

37. K. Paragas, S. Vissapragada, H. A. Knutson, A. Oklopčić, Y. Chachan, M. Greklek-Mc Keon, F. Dai, S. Tinyanont, G. Vasisht, Metastable helium reveals an extended atmosphere for the gas giant HAT-P-18b. *Astrophys. J. Lett.* **909**, L10 (2021).

38. C. A. L. Bailer-Jones, J. Rybizki, M. Fouesneau, M. Demleitner, R. Andrae, Estimating distances from parallaxes. V. Geometric and photogeometric distances to 1.47 billion stars in Gaia early data release 3. *Astron. J.* **161**, 147–170 (2021).

39. D. J. A. Brown, Discrepancies between isochrone fitting and gyrochronology for exoplanet host stars? *Mon. Notices Royal Astron. Soc.* **442**, 1844–1862 (2014).

40. H. A. Knutson, B. J. Fulton, B. T. Montet, M. Kao, H. Ngo, A. W. Howard, J. R. Crepp, S. Hinkley, G. Á. Bakos, K. Batygin, J. A. Johnson, T. D. Morton, P. S. Muirhead, Friends of hot jupiters. I. A radial velocity search for massive, long-period companions to close-in gas giant planets. *Astrophys. J.* **785**, 126–148 (2014).

41. M. Zhao, J. G. O'Rourke, J. T. Wright, H. A. Knutson, A. Burrows, J. Fortney, H. Ngo, B. J. Fulton, C. Baranec, R. Riddle, N. M. Law, P. S. Muirhead, S. Hinkley, A. P. Showman, J. Curtis, R. Burruss, Characterization of the atmosphere of the hot Jupiter HAT-P-32Ab and the M-dwarf companion HAT-P-32B. *Astrophys. J.* **796**, 115–129 (2014).

42. J. J. Fortney, M. S. Marley, J. W. Barnes, Planetary radii across five orders of magnitude in mass and stellar insolation: Application to transits. *Astrophys. J.* **659**, 1661–1672 (2007).

43. S. Albrecht, J. N. Winn, J. A. Johnson, A. W. Howard, G. W. Marcy, R. P. Butler, P. Arriagada, J. D. Crane, S. A. Shectman, I. B. Thompson, T. Hirano, G. Bakos, J. D. Hartman, Obliquities of hot Jupiter host stars: Evidence for tidal interactions and primordial misalignments. *Astron. J.* **757**, 18–42 (2012).

44. E. R. Adams, A. K. Dupree, C. Kulesa, D. McCarthy, Adaptive optics images. II. 12 Kepler objects of interest and 15 confirmed transiting planets. *Astron. J.* **146**, 9 (2013).

45. H. Ngo, H. A. Knutson, S. Hinkley, J. R. Crepp, E. B. Bechter, K. Batygin, A. W. Howard, J. A. Johnson, T. D. Morton, P. S. Muirhead, Friends of hot Jupiters. II. No correspondence between hot-Jupiter spin-orbit misalignment and the incidence of directly imaged stellar companions. *Astrophys. J.* **800**, 138–159 (2015).

46. D. Krolikowski, "Exploring the formation and evolutionary pathways of young stars and planetary systems at high precision," thesis, University of Texas at Austin (2022).

47. E. Oliva, L. Origlia, S. Scuderi, S. Benatti, I. Carleo, E. Lapenna, A. Mucciarelli, C. Baffa, V. Biliotti, L. Carbonaro, G. Falcini, E. Giani, M. Iuzzolino, F. Massi, N. Sanna, M. Sozzi, A. Tozzi, A. Ghedina, F. Ghinassi, M. Lodi, A. Harutyunyan, M. P. I. Arcetri, I. Bologna, Inaf-Catania, I. Padova U. Bologna, I. G. Galilei, Lines and continuum sky emission in the near infrared: Observational constraints from deep high spectral resolution spectra with GIANO-TNG. *Astron. Astrophys.* **581**, 10.1051/0004-6361/201526291 (2015).

48. Q. H. Tran, B. P. Bowler, W. D. Cochran, M. Endl, G. Stefánsson, S. Mahadevan, J. P. Ninan, C. F. Bender, S. Halverson, A. Roy, R. C. Terrien, The epoch of giant planet migration planet search

program. I. Near-infrared radial velocity jitter of young sun-like stars. *Astron. J.* **161**, 173–189 (2021).

49. Astropy Collaboration, T. P. Robitaille, E. J. Tollerud, P. Greenfield, M. Droettboom, E. Bray, T. Aldcroft, M. Davis, A. Ginsburg, A. M. Price-Whelan, W. E. Kerzendorf, A. Conley, N. Crighton, K. Barbary, D. Muna, H. Ferguson, F. Grollier, M. M. Parikh, P. H. Nair, H. M. Günther, C. Deil, J. Woillez, S. Conseil, R. Kramer, J. E. H. Turner, L. Singer, R. Fox, B. A. Weaver, V. Zabalza, Z. I. Edwards, K. Azalee Bostroem, D. J. Burke, A. R. Casey, S. M. Crawford, N. Dencheva, J. Ely, T. Jenness, K. Labrie, P. L. Lim, F. Pierfederici, A. Pontzen, A. Ptak, B. Refsdal, M. Servillat, O. Streicher, Astropy: A community Python package for astronomy. *Astron. Astrophys.* **558**, 33–41 (2013).

50. The Astropy Collaboration, A. M. Price-Whelan, B. M. Sipőcz, H. M. Günther, P. L. Lim, S. M. Crawford, S. Conseil, D. L. Shupe, M. W. Craig, N. Dencheva, A. Ginsburg, J. T. VanderPlas, L. D. Bradley, D. Pérez-Suárez, M. de Val-Borro; Primary Paper Contributors, T. L. Aldcroft, K. L. Cruz, T. P. Robitaille, E. J. Tollerud; Astropy Coordination Committee, C. Ardelean, T. Babej, Y. P. Bach, M. Bachetti, A. V. Bakanov, S. P. Bamford, G. Barentsen, P. Barmby, A. Baumbach, K. L. Berry, F. Biscani, M. Boquien, K. A. Bostroem, L. G. Bouma, G. B. Brammer, E. M. Bray, H. Breytenbach, H. Buddelmeijer, D. J. Burke, G. Calderone, J. L. Cano Rodríguez, M. Cara, J. V. M. Cardoso, S. Cheedella, Y. Copin, L. Corrales, D. Crichton, D. D'Avella, C. Deil, É. Depagne, J. P. Dietrich, A. Donath, M. Droettboom, N. Earl, T. Erben, S. Fabbro, L. A. Ferreira, T. Finethy, R. T. Fox, L. H. Garrison, S. L. J. Gibbons, D. A. Goldstein, R. Gommers, J. P. Greco, P. Greenfield, A. M. Groener, F. Grollier, A. Hagen, P. Hirst, D. Homeier, A. J. Horton, G. Hosseinzadeh, L. Hu, J. S. Hunkeler, Ž. Ivezić, A. Jain, T. Jenness, G. Kanarek, S. Kendrew, N. S. Kern, W. E. Kerzendorf, A. Khvalko, J. King, D. Kirkby, A. M. Kulkarni, A. Kumar, A. Lee, D. Lenz, S. P. Littlefair, Z. Ma, D. M. Macleod, M. Mastropietro, C. McCully, S. Montagnac, B. M. Morris, M. Mueller, S. J. Mumford, D. Muna, N. A. Murphy, S. Nelson, G. H. Nguyen, J. P. Ninan, M. Nöthe, S. Ogaz, S. Oh, J. K. Parejko, N. Parley, S. Pascual, R. Patil, A. A. Patil, A. L. Plunkett, J. X. Prochaska, T. Rastogi, V. Reddy Janga, J. Sabater, P. Sakurikar, M. Seifert, L. E. Sherbert, H. Sherwood-Taylor, A. Y. Shih, J. Sick, M. T. Silbiger, S. Singanamalla, L. P. Singer, P. H. Sladen, K. A. Sooley, S. Sornarajah, O. Streicher, P. Teuben, S. W. Thomas, G. R. Tremblay, J. E. H. Turner, V. Terrón, M. H. van Kerkwijk, A. de la Vega, L. L. Watkins, B. A. Weaver, J. B. Whitmore, J. Woillez, V. Zabalza; Astropy Contributors,

The astropy project: Building an open-science project and status of the v2.0 core package. *Astron. J.* **156**, 123–141 (2018).

51. M. Zechmeister, A. Reiners, P. J. Amado, M. Azzaro, F. F. Bauer, V. J. S. Béjar, J. A. Caballero, E. W. Guenther, H.-J. Hagen, S. V. Jeffers, A. Kaminski, M. Kürster, R. Launhardt, D. Montes, J. C. Morales, A. Quirrenbach, S. Reffert, I. Ribas, W. Seifert, L. Tal-Or, V. Wolthoff, Spectrum radial velocity analyser (SERVAL). High-precision radial velocities and two alternative spectral indicators. *Astron. Astrophys.* **609**, 12–24 (2018).

52. J. Martin, B. Fuhrmeister, M. Mittag, T. O. B. Schmidt, A. Hempelmann, J. N. González-Pérez, J. H. M. M. Schmitt, The Ca II infrared triplet's performance as an activity indicator compared to Ca II H and K. Empiricalrelations to convert Ca II infrared triplet measurements to common activity indices. *Astron. Astrophys.* **605**, 113–127 (2017).

53. M. K. Alam, M. López-Morales, N. Nikolov, D. K. Sing, G. W. Henry, C. Baxter, J. M. Désert, J. K. Barstow, T. Mikal-Evans, V. Bourrier, P. Lavvas, H. R. Wakeford, M. H. Williamson, J. Sanz-Forcada, L. A. Buchhave, O. Cohen, A. G. Muñoz, The Hubble space telescope PanCET program: An optical to infrared transmission spectrum of HAT-P-32Ab. *Astron. J.* **160**, 51–69 (2020).

54. J. M. Stone, K. Tomida, C. J. White, K. G. Felker, The Athena++ adaptive mesh refinement framework: Design and magnetohydrodynamic solvers. *Astrophys. J. Suppl. Ser.* **249**, 4–43 (2020).

55. D. Linssen, A. Oklopčić, M. MacLeod, Constraining planetary mass-loss rates by simulating Parker wind profiles with Cloudy. *Astron. Astrophys.* **667**, 54–67 (2022).

56. B. E. Wood, H.-R. Müller, G. P. Zank, J. L. Linsky, Measured mass-loss rates of solar-like stars as a function of age and activity. *Astrophys. J.* **574**, 412–425 (2002).

57. J. Sanz-Forcada, G. Micela, I. Ribas, A. M. T. Pollock, C. Eiroa, A. Velasco, E. Solano, D. García-Álvarez, Estimation of the XUV radiation onto close planets and their evaporation. *Astron. Astrophys.* **532**, 6–23 (2011).

58. A. Oklopčić, C. M. Hirata, A new window into escaping exoplanet atmospheres: 10830 Å line of helium. *Astrophys. J. Lett.* **855**, L11 (2018).

59. J. P. Ninan, G. Stefansson, S. Mahadevan, C. Bender, P. Robertson, L. Ramsey, R. Terrien, J. Wright, S. A. Diddams, S. Kanodia, W. Cochran, M. Endl, E. B. Ford, C. Fredrick, S. Halverson, F. Hearty, J. Jennings, K. Kaplan, E. Lubar, A. J. Metcalf, A. Monson, C. Nitroy, A. Roy, C. Schwab, Evidence for He I 10830 Å absorption during the transit of a warm Neptune around the M-dwarf GJ 3470 with the Habitable-zone Planet Finder. *Astrophys. J.* **894**, 97–105 (2020).

60. M. Zhang, H. A. Knutson, F. Dai, L. Wang, G. R. Ricker, R. P. Schwarz, C. Mann, K. Collins, Detection of atmospheric escape from four young mini-Neptunes. *Astron. J.* **165**, 62–77 (2023).

61. E. Gaidos, T. Hirano, R. A. Lee, H. Harakawa, K. Hodapp, S. Jacobson, T. Kotani, T. Kudo, T. Kurokawa, M. Kuzuhara, J. Nishikawa, M. Omiya, T. Serizawa, M. Tamura, A. Ueda, S. Vievard, Planet(esimal)s around stars with *TESS* (PAST) III: A search for triplet He I in the atmospheres of two 200 Myr-old planets. *Mon. Notices. Royal Astron. Soc.* **518**, 3777–3783 (2022).

62. A. W. Mann, A. Vanderburg, A. C. Rizzuto, A. L. Kraus, P. Berlind, A. Bieryla, M. L. Calkins, G. A. Esquerdo, D. W. Latham, G. N. Mace, N. R. Morris, S. N. Quinn, K. R. Sokal, R. P. Stefanik, Zodiacal exoplanets in time (ZEIT). VI. A three-planet system in the Hyades cluster including an Earth-sized planet. *Astron. J.* **155**, 4–14 (2018).

63. D. R. Ciardi, I. J. M. Crossfield, A. D. Feinstein, J. E. Schlieder, E. A. Petigura, T. J. David, M. Bristow, R. I. Patel, L. Arnold, B. Benneke, J. L. Christiansen, C. D. Dressing, B. J. Fulton, A. W. Howard, H. Isaacson, E. Sinukoff, B. Thackeray, K2-136: A binary system in the Hyades cluster hosting a Neptune-sized planet. *Astron. J.* **155**, 10–20 (2018).

64. E. Gaidos, T. Hirano, M. Omiya, M. Kuzuhara, T. Kotani, M. Tamura, H. Harakawa, T. Kudo, Zodiacal exoplanets in time (ZEIT). XIV. He I transit spectroscopy of the 650 Myr Hyades planet K2-136c. *Res. Notes AAS* **5**, 238 (2021).

65. J. Fernández, P. J. Wheatley, X-ray irradiation of three planets around Hyades star K2–136. *Astron. Notes* **343**, e210076 (2022).

66. O. Barragán, S. Aigrain, D. Kubyshkina, D. Gandolfi, J. Livingston, M. C. V. Fridlund, L. Fossati, J. Korth, H. Parviainen, L. Malavolta, E. Palle, H. J. Deeg, G. Nowak, V. M. Rajpaul, N. Zicher, G.

Antoniciello, N. Narita, S. Albrecht, L. R. Bedin, J. Cabrera, W. D. Cochran, J. de Leon, P. Eigmüller, A. Fukui, V. Granata, S. Grziwa, E. Guenther, A. P. Hatzes, N. Kusakabe, D. W. Latham, M. Libralato, R. Luque, P. Montañés-Rodríguez, F. Murgas, D. Nardiello, I. Pagano, G. Piotto, C. M. Persson, S. Redfield, M. Tamura, Radial velocity confirmation of K2-100b: A young, highly irradiated, and low-density transiting hot Neptune. *Mon. Notices Royal Astron. Soc.* **490**, 698–708 (2019).

67. E. Gaidos, T. Hirano, A. W. Mann, D. A. Owens, T. A. Berger, K. France, A. Vanderburg, H. Harakawa, K. W. Hodapp, M. Ishizuka, S. Jacobson, M. Konishi, T. Kotani, T. Kudo, T. Kurokawa, M. Kuzuhara, J. Nishikawa, M. Omiya, T. Serizawa, M. Tamura, A. Ueda, Zodiacal exoplanets in time - X. The orbit and atmosphere of the young ‘neptune desert’-dwelling planet K2-100b. *Mon. Notices Royal Astron. Soc.* **495**, 650–662 (2020).

68. T. J. David, E. A. Petigura, R. Luger, D. Foreman-Mackey, J. H. Livingston, E. E. Mamajek, L. A. Hillenbrand, Four newborn planets transiting the young solar analog V1298 tau. *Astrophys. J. Lett.* **885**, L12 (2019).

69. S. Vissapragada, G. Stefánsson, M. Greklek-McKeon, A. Oklopčić, H. A. Knutson, J. P. Ninan, S. Mahadevan, C. I. Cañas, Y. Chachan, W. D. Cochran, K. A. Collins, F. Dai, T. J. David, S. Halverson, S. L. Hawley, L. Hebb, S. Kanodia, A. F. Kowalski, J. H. Livingston, M. Maney, A. J. Metcalf, C. Morley, L. W. Ramsey, P. Robertson, A. Roy, J. Spake, C. Schwab, R. C. Terrien, S. Tinyanont, G. Vasisht, J. Wisniewski, A search for planetary metastable helium absorption in the V1298 tau system. *Astron. J.* **162**, 222–231 (2021).

70. K. Poppenhaeger, L. Ketzer, M. Mallonn, X-ray irradiation and evaporation of the four young planets around V1298 tau. *Mon. Notices Royal Astron. Soc.* **500**, 4560–4572 (2020).

71. M. Gillon, A. H. M. J. Triaud, B. O. Demory, E. Jehin, E. Agol, K. M. Deck, S. M. Lederer, J. de Wit, A. Burdanov, J. G. Ingalls, E. Bolmont, J. Leconte, S. N. Raymond, F. Selsis, M. Turbet, K. Barkaoui, A. Burgasser, M. R. Burleigh, S. J. Carey, A. Chaushev, C. M. Copperwheat, L. Delrez, C. S. Fernandes, D. L. Holdsworth, E. J. Kotze, V. van Grootel, Y. Almleaky, Z. Benkhaldoun, P. Magain, D. Queloz, Seven temperate terrestrial planets around the nearby ultracool dwarf star TRAPPIST-1. *Nature* **542**, 456–460 (2017).

72. P. J. Wheatley, T. Louden, V. Bourrier, D. Ehrenreich, M. Gillon, Strong XUV irradiation of the Earth-sized exoplanets orbiting the ultracool dwarf TRAPPIST-1. *Mon. Notices Royal Astron. Soc.* **465**, L74–78 (2017).

73. V. Krishnamurthy, T. Hirano, G. Stefánsson, J. P. Ninan, S. Mahadevan, E. Gaidos, R. Kopparapu, B. Sato, Y. Hori, C. F. Bender, C. I. Cañas, S. A. Diddams, S. Halverson, H. Harakawa, S. Hawley, F. Hearty, L. Hebb, K. Hodapp, S. Jacobson, S. Kanodia, M. Konishi, T. Kotani, A. Kowalski, T. Kudo, T. Kurokawa, M. Kuzuhara, A. Lin, M. Maney, A. J. Metcalf, B. Morris, J. Nishikawa, M. Omiya, P. Robertson, A. Roy, C. Schwab, T. Serizawa, M. Tamura, A. Ueda, S. Vievard, J. Wisniewski, Nondetection of helium in the upper atmospheres of TRAPPIST-1b, e, and f. *Astron. J.* **162**, 82–89 (2021).

74. E. Agol, C. Dorn, S. L. Grimm, M. Turbet, E. Ducrot, L. Delrez, M. Gillon, B. O. Demory, A. Burdanov, K. Barkaoui, Z. Benkhaldoun, E. Bolmont, A. Burgasser, S. Carey, J. de Wit, D. Fabrycky, D. Foreman-Mackey, J. Haldemann, D. M. Hernandez, J. Ingalls, E. Jehin, Z. Langford, J. Leconte, S. M. Lederer, R. Luger, R. Malhotra, V. S. Meadows, B. M. Morris, F. J. Pozuelos, D. Queloz, S. N. Raymond, F. Selsis, M. Sestovic, A. H. M. J. Triaud, V. V. Grootel, Refining the transit-timing and photometric analysis of TRAPPIST-1: Masses, radii, densities, dynamics, and ephemerides. *Planet. Sci. J.* **2**, 1–38 (2021).

75. O. D. Turner, D. R. Anderson, K. Barkaoui, F. Bouchy, Z. Benkhaldoun, D. J. A. Brown, A. Burdanov, A. Collier Cameron, E. Ducrot, M. Gillon, C. Hellier, E. Jehin, M. Lendl, P. F. L. Maxted, L. D. Nielsen, F. Pepe, D. Pollacco, F. J. Pozuelos, D. Queloz, D. Ségransan, B. Smalley, A. H. M. J. Triaud, S. Udry, R. G. West, Three hot-Jupiters on the upper edge of the mass-radius distribution: WASP-177, WASP-181, and WASP-183. *Mon. Notices Royal Astron. Soc.* **485**, 5790–5799 (2019).

76. J. Kirk, L. A. dos Santos, M. López-Morales, M. K. Alam, A. Oklopčić, M. MacLeod, L. Zeng, G. Zhou, Keck/NIRSPEC studies of He I in the atmospheres of two inflated hot gas giants orbiting K dwarfs: WASP-52b and WASP-177b. *Astron. J.* **164**, 24–37 (2022).

77. D. Kasper, J. L. Bean, A. Oklopčić, I. Malsky, E. M. R. Kempton, J. M. Désert, L. A. Rogers, M. Mansfield, Nondetection of helium in the upper atmospheres of three sub-Neptune exoplanets. *Astron. J.* **160**, 258–270 (2020).

78. K. Rice, L. Malavolta, A. Mayo, A. Mortier, L. A. Buchhave, L. Affer, A. Vanderburg, M. Lopez-Morales, E. Poretti, L. Zeng, A. C. Cameron, M. Damasso, A. Coffinet, D. W. Latham, A. S. Bonomo, F. Bouchy, D. Charbonneau, X. Dumusque, P. Figueira, A. F. Martinez Fiorenzano, R. D. Haywood, J. A. Johnson, E. Lopez, C. Lovis, M. Mayor, G. Micela, E. Molinari, V. Nascimbeni, C. Nava, F. Pepe, D. F. Phillips, G. Piotto, D. Sasselov, D. Ségransan, A. Sozzetti, S. Udry, C. Watson, Masses and radii for the three super-Earths orbiting GJ 9827, and implications for the composition of small exoplanets. *Mon. Notices Royal Astron. Soc.* **484**, 3731–3745 (2019).

79. T. G. Ellis, T. Boyajian, K. von Braun, R. Ligi, D. Mourard, D. Dragomir, G. H. Schaefer, C. D. Farrington, Directly determined properties of HD 97658 from interferometric observations. *Astron. J.* **162**, 118–126 (2021).

80. B.-O. Demory, M. Gillon, D. Deming, D. Valencia, S. Seager, B. Benneke, C. Lovis, P. Cubillos, J. Harrington, K. B. Stevenson, M. Mayor, F. Pepe, D. Queloz, D. Ségransan, S. Udry, Detection of atransit of the super-Earth 55 Cancri e with warm Spitzer. *Astron. Astrophys.* **533**, 114–120 (2011).

81. V. Bourrier, X. Dumusque, C. Dorn, G. W. Henry, N. Astudillo-Defru, J. Rey, B. Benneke, G. Hébrard, C. Lovis, B. O. Demory, C. Moutou, D. Ehrenreich, The 55 Cancri system reassessed. *Astron. Astrophys.* **619**, 1–18 (2018).

82. M. Zhang, H. A. Knutson, L. Wang, F. Dai, A. Oklopcic, R. Hu, No escaping helium from 55 Cnc e. *Astron. J.* **161**, 181–198 (2021).

83. B. S. Gaudi, K. G. Stassun, K. A. Collins, T. G. Beatty, G. Zhou, D. W. Latham, A. Bieryla, J. D. Eastman, R. J. Siverd, J. R. Crepp, E. J. Gonzales, D. J. Stevens, L. A. Buchhave, J. Pepper, M. C. Johnson, K. D. Colon, E. L. N. Jensen, J. E. Rodriguez, V. Bozza, S. C. Novati, G. D'Ago, M. T. Dumont, T. Ellis, C. Gaillard, H. Jang-Condell, D. H. Kasper, A. Fukui, J. Gregorio, A. Ito, J. F. Kielkopf, M. Manner, K. Matt, N. Narita, T. E. Oberst, P. A. Reed, G. Scarpetta, D. C. Stephens, R. R. Yeigh, R. Zambelli, B. J. Fulton, A. W. Howard, D. J. James, M. Penny, D. Bayliss, I. A. Curtis, D. L. DePoy, G. A. Esquerdo, A. Gould, M. D. Joner, R. B. Kuhn, J. Labadie-Bartz, M. B. Lund, J. L. Marshall, K. K. McLeod, R. W. Pogge, H. Relles, C. Stockdale, T. G. Tan, M. Trueblood, P. Trueblood, A giant planet undergoing extreme-ultraviolet irradiation by its hot massive-star host. *Nature* **546**, 514–518 (2017).

84. G. Torres, J. N. Winn, M. J. Holman, Improved parameters for extrasolar transiting planets. *Astrophys. J.* **677**, 1324–1342 (2008).

85. G. Maciejewski, A. Niedzielski, G. Nowak, E. Pallé, B. Tingley, R. Errmann, R. Neuhäuser, On the GJ 436 planetary system. *Acta Astron.* **64**, 323–335 (2014).

86. A. H. M. J. Triaud, M. Gillon, D. Ehrenreich, E. Herrero, M. Lendl, D. R. Anderson, A. Collier Cameron, L. Delrez, B. O. Demory, C. Hellier, K. Heng, E. Jehin, P. F. L. Maxted, D. Pollacco, D. Queloz, I. Ribas, B. Smalley, A. M. S. Smith, S. Udry, WASP-80b has a dayside within the T-dwarf range. *Mon. Notices Royal Astron. Soc.* **450**, 2279–2290 (2015).

87. L. Fossati, G. Guilluy, I. F. Shaikhislamov, I. Carleo, F. Borsa, A. S. Bonomo, P. Giacobbe, M. Rainer, C. Cecchi-Pestellini, M. L. Khodachenko, M. A. Efimov, M. S. Rumenskikh, I. B. Miroshnichenko, A. G. Berezutsky, V. Nascimbeni, M. Brogi, A. F. Lanza, L. Mancini, L. Affer, S. Benatti, K. Biazzo, A. Bignamini, D. Carosati, R. Claudi, R. Cosentino, E. Covino, S. Desidera, A. Fiorenzano, A. Harutyunyan, A. Maggio, L. Malavolta, J. Maldonado, G. Micela, E. Molinari, I. Pagano, M. Pedani, G. Piotto, E. Poretti, G. Scandariato, A. Sozzetti, H. Stoev, The GAPS Programme at TNG. XXXII. The revealing non-detection of metastable He I in the atmosphere of the hot Jupiter WASP-80b. *Astron. Astrophys.* **658**, 136–149 (2022).

88. L. A. dos Santos, D. Ehrenreich, V. Bourrier, R. Allart, G. King, M. Lendl, C. Lovis, S. Margheim, J. Mel'endez, J. Seidel, S. Sousa, Search for helium in the upper atmosphere of the hot Jupiter WASP-127 b using Gemini/Phoenix. *Astron. Astrophys.* **640**, 29–33 (2020).

89. K. W. F. Lam, F. Faedi, D. J. A. Brown, D. R. Anderson, L. Delrez, M. Gillon, G. Hébrard, M. Lendl, L. Mancini, J. Southworth, B. Smalley, A. H. M. Triaud, O. D. Turner, K. L. Hay, D. J. Armstrong, S. C. C. Barros, A. S. Bonomo, F. Bouchy, P. Boumis, A. Collier Cameron, A. P. Doyle, C. Hellier, T. Henning, E. Jehin, G. King, J. Kirk, T. Louden, P. F. L. Maxted, J. J. Mc Cormac, H. P. Osborn, E. Palle, F. Pepe, D. Pollacco, J. Prieto-Arranz, D. Queloz, J. Rey, D. Ségransan, S. Udry, S. Walker, R. G. West, P. J. Wheatley, From dense hot Jupiter to low-density Neptune: The discovery of WASP-127b, WASP-136b, and WASP-138b. *Astron. Astrophys.* **599**, A3 (2017).

90. J. V. Seidel, M. Lendl, V. Bourrier, D. Ehrenreich, R. Allart, S. G. Sousa, H. M. Cegla, X. Bonfils, U. Conod, A. Grandjean, A. Wyttenbach, N. Astudillo-Defru, D. Bayliss, K. Heng, B. Lavie, C. Lovis, C. Melo, F. Pepe, D. Ségransan, S. Udry, Hot exoplanet atmospheres resolved with transit spectroscopy (HEARTS). VI. Non-detection of sodium with HARPS on the bloated super-Neptune WASP-127b. *Astron. Astrophys.* **643**, A45–54 (2020).

91. N. Casasayas-Barris, J. Orell-Miquel, M. Stangret, L. Nortmann, F. Yan, M. Oshagh, E. Palle, J. Sanz-Forcada, M. López-Puertas, E. Nagel, R. Luque, G. Morello, I. A. G. Snellen, M. Zechmeister, A. Quirrenbach, J. A. Caballero, I. Ribas, A. Reiners, P. J. Amado, G. Bergond, S. Czesla, T. Henning, S. Khalafinejad, K. Molaverdikhani, D. Montes, M. Perger, A. Sánchez-López, E. Sedaghati, CARMENES detection of the ca II infrared triplet and possible evidence of He I in the atmosphere of WASP-76b. *Astron. Astrophys.* **654**, A163–182 (2021).

92. R. G. West, J.-M. Almenara, D. R. Anderson, F. Bouchy, D. J. A. Brown, A. Collier Cameron, M. Deleuil, L. Delrez, A. P. Doyle, F. Faedi, A. Fumel, M. Gillon, G. Hebrard, C. Hellier, E. Jehin, M. Lendl, P. F. L. Maxted, F. Pepe, D. Pollacco, D. Queloz, D. Segransan, B. Smalley, A. M. S. Smith, A. H. M. J. Triaud, S. Udry, Three irradiated and bloated hot Jupiters: WASP-76b, WASP-82b, and WASP-90b. *Astron. Astrophys.* **585**, A126–132 (2016).

93. J. D. Hartman, G. Á. Bakos, B. Sato, G. Torres, R. W. Noyes, D. W. Latham, G. Kovács, D. A. Fischer, A. W. Howard, J. A. Johnson, G. W. Marcy, L. A. Buchhave, G. Füresz, G. Perumpilly, B. Béky, R. P. Stefanik, D. D. Sasselov, G. A. Esquerdo, M. Everett, Z. Csubry, J. Lázár, I. Papp, P. Sári, HAT-P-18b and HAT-P-19b: Two low-density Saturn-mass planets transiting metal-rich K stars. *Astrophys. J.* **726**, 52–67 (2011).

94. O. Barragán, D. J. Armstrong, D. Gandolfi, I. Carleo, A. A. Vidotto, C. Villarreal D'Angelo, A. Oklopčić, H. Isaacson, D. Oddo, K. Collins, M. Fridlund, S. G. Sousa, C. M. Persson, C. Hellier, S. Howell, A. Howard, S. Redfield, N. Eisner, I. Y. Georgieva, D. Dragomir, D. Bayliss, L. D. Nielsen, B. Klein, S. Aigrain, M. Zhang, J. Teske, J D. Twicken, J. Jenkins, M. Esposito, V. Van Eylen, F. Rodler, V. Adibekyan, J. Alarcon, D. R. Anderson, J. M. Akana Murphy, D. Barrado, S. C. C. Barros, B. Benneke, F. Bouchy, E. M. Bryant, P. Butler, J. Burt, J. Cabrera, S. Casewell, P. Chaturvedi, R. Cloutier, W. D. Cochran, J. Crane, I. Crossfield, N. Crouzet, K. I. Collins, F. Dai, H. J. Deeg, A.

Deline, O. D. S. Demangeon, X. Dumusque, P. Figueira, E. Furlan, C. Gnilka, M. R. Goad, E. Goffo, F. Gutiérrez-Canales, A. Hadjigeorghiou, Z. Hartman, A. P. Hatzes, M. Harris, B. Henderson, T. Hirano, S. Hojjatpanah, S. Hoyer, P. Kabáth, J. Korth, J. Lillo-Box, R. Luque, M. Marmier, T. Močnik, A. Muresan, F. Murgas, E. Nagel, H. L. M. Osborne, A. Osborn, H. P. Osborn, E. Palle, M. Raimbault, G. R. Ricker, R. A. Rubenzahl, C. Stockdale, N. C. Santos, N. Scott, R. P. Schwarz, S. Shectman, M. Raimbault, S. Seager, D. Ségransan, L. M. Serrano, M. Skarka, A. M. S. Smith, J. Šubjak, T. G. Tan, S. Udry, C. Watson, P. J. Wheatley, R. West, J. N. Winn, S. X. Wang, A. Wolfgang, C. Ziegler, The young HD 73583 (TOI-560) planetary system: Two 10-$M_\oplus$ mini-Neptunes transiting a 500-Myr-old, bright, and active K dwarf. *Mon. Notices Royal Astron. Soc.* **514**, 1606–1627 (2022).

95. K. G. Stassun, K. A. Collins, B. S. Gaudi, Accurate empirical radii and masses of planets and their host stars with *Gaia* parallaxes. *Astron. J.* **153**, 136–155 (2017).

96. F. J. Alonso-Floriano, I. Snellen, S. Czesla, F. Bauer, M. Salz, M. Lamp'on, L. Lara, E. Nagel, M. L'opez-Puertas, L. Nortmann, A. Sánchez-López, J. Sanz-Forcada, J. Caballero, A. Reiners, I. Ribas, A. Quirrenbach, P. Amado, J. Aceituno, G. Anglada-Escud'e, V. B'ejar, M. Brinkmöller, A. Hatzes, T. Henning, A. Kaminski, M. Kürster, F. Labarga, D. Montes, E. Pall'e, J. Schmitt, M. Z. Zapatero Osorio, He I λ 10 830 Å in the transmission spectrum of HD209458 b. *Astron. Astrophys.* **629**, 110–116 (2019).

97. H. P. Osborn, A. Bonfanti, D. Gandolfi, C. Hedges, A. Leleu, A. Fortier, D. Futyan, P. Gutermann, P. F. L. Maxted, L. Borsato, K. A. Collins, J. Gomes da Silva, Y. Gómez Maqueo Chew, M. J. Hooton, M. Lendl, H. Parviainen, S. Salmon, N. Schanche, L. M. Serrano, S. G. Sousa, A. Tuson, S. Ulmer-Moll, V. Van Grootel, R. D. Wells, T. G. Wilson, Y. Alibert, R. Alonso, G. Anglada, J. Asquier, D. B. Y. Navascues, W. Baumjohann, T. Beck, W. Benz, F. Biondi, X. Bonfils, F. Bouchy, A. Brandeker, C. Broeg, T. Bárczy, S. C. C. Barros, J. Cabrera, S. Charnoz, A. C. Cameron, S. Csizmadia, M. B. Davies, M. Deleuil, L. Delrez, B.-O. Demory, D. Ehrenreich, A. Erikson, L. Fossati, M. Fridlund, M. Gillon, M. A. Gómez-Muñoz, M. Güdel, K. Heng, S. Hoyer, K. G. Isaak, L. Kiss, J. Laskar, A. L. Des Etangs, C. Lovis, D. Magrin, L. Malavolta, J. M. Cormac, V. Nascimbeni, G. Olofsson, R. Ottensamer, I. Pagano, E. Pallé, G. Peter, D. Piazza, G. Piotto, D. Pollacco, D. Queloz, R. Ragazzoni, N. Rando, H. Rauer, C. Reimers, I. Ribas, O. D. S. Demangeon, A. M. S.

Smith, L. Sabin, N. Santos, G. Scandariato, U. Schroffenegger, R. P. Schwarz, A. Shporer, A. E. Simon, M. Steller, G. M. Szabó, D. Ségransan, N. Thomas, S. Udry, I. Walter, N. Walton, Uncovering the true periods of the young sub-Neptunes orbiting TOI-2076. *Astron. Astrophys.* **644**, 156–172 (2022).

98. M. Salz, S. Czesla, P. Schneider, E. Nagel, J. Schmitt, L. Nortmann, F. J. Alonso-Floriano, M. L'opez-Puertas, M. Lamp'on, F. Bauer, I. Snellen, E. Pall'e, J. Caballero, F. Yan, G. Chen, J. Sanz-Forcada, P. Amado, A. Quirrenbach, I. Ribas, A. Reiners, V. B'ejar, N. Casasayas-Barris, M. Cort'es-Contreras, S. Dreizler, E. Guenther, T. Henning, S. Jeffers, A. Kaminski, M. Kürster, M. Lafarga, L. Lara, K. Molaverdikhani, D. Montes, J. Morales, A. Sánchez-López, W. Seifert, M. Z. Zapatero Osorio, M. Zechmeister, Detection of He I λ10830 Å absorption on HD 189733 b with CARMENES high-resolution transmission spectroscopy. *Astron. Astrophys.* **620**, 97–109 (2018).

99. G. Guilluy, V. Andretta, F. Borsa, P. Giacobbe, A. Sozzetti, E. Covino, V. Bourrier, L. Fossati, A. S. Bonomo, M. Esposito, M. S. Giampapa, A. Harutyunyan, M. Rainer, M. Brogi, G. Bruno, R. Claudi, G. Frustagli, A. F. Lanza, L. Mancini, L. Pino, E. Poretti, G. Scandariato, L. Affer, C. Baffa, A. Baruffolo, S. Benatti, K. Biazzo, A. Bignamini, W. Boschin, I. Carleo, M. Cecconi, R. Cosentino, M. Damasso, S. Desidera, G. Falcini, A. F. Martinez Fiorenzano, A. Ghedina, E. González-Álvarez, J. Guerra, N. Hernandez, G. Leto, A. Maggio, L. Malavolta, J. Maldonado, G. Micela, E. Molinari, V. Nascimbeni, I. Pagano, M. Pedani, G. Piotto, A. Reiners, The GAPS programme at TNG. XXII. The GIARPS view of the extended helium atmosphere of HD 189733 b accounting for stellar activity. *Astron. Astrophys.* **639**, A49 (2020).

100. G. Á. Bakos, G. Torres, A. Pál, J. Hartman, G. Kovács, R. W. Noyes, D. W. Latham, D. D. Sasselov, B. Sipőcz, G. A. Esquerdo, D. A. Fischer, J. A. Johnson, G. W. Marcy, R. P. Butler, H. Isaacson, A. Howard, S. Vogt, G. Kovács, J. Fernandez, A. Moór, R. P. Stefanik, J. Lázár, I. Papp, P. Sári, HAT-P-11b: A super-Neptune planet transiting a bright K star in the Kepler field. *Astrophys. J.* **710**, 1724–1745 (2010).

101. S. W. Yee, E. A. Petigura, B. J. Fulton, H. A. Knutson, K. Batygin, G. Bakos, J. D. Hartman, L. A. Hirsch, A. W. Howard, H. Isaacson, M. R. Kosiarek, E. Sinukoff, L. M. Weiss, HAT-P-11: Discovery of a second planet and a clue to understanding exoplanet obliquities. *Astron. J.* **155**, 255–267 (2018).

102. S. Awiphan, E. Kerins, S. Pichadee, S. Komonjinda, V. S. Dhillon, W. Rujopakarn, S. Poshyachinda, T. R. Marsh, D. E. Reichart, K. M. Ivarsen, J. B. Haislip, Transit timing variation and transmission spectroscopy analyses of the hot Neptune GJ3470b. *Mon. Notices Royal Astron. Soc.* **463**, 2574–2582 (2016).

103. E. Palle, L. Nortmann, N. Casasayas-Barris, M. Lampón, M. López-Puertas, J. A. Caballero, J. Sanz-Forcada, L. M. Lara, E. Nagel, F. Yan, F. J. Alonso-Floriano, P. J. Amado, G. Chen, C. Cifuentes, M. Cortés-Contreras, S. Czesla, K. Molaverdikhani, D. Montes, V. M. Passegger, A. Quirrenbach, A. Reiners, I. Ribas, A. Sánchez-López, A. Schweitzer, M. Stangret, M. R. Zapatero Osorio, M. Zechmeister, AHe I upper atmosphere around the warm Neptune GJ 3470 b. *Astron. Astrophys.* **638**, 61–68 (2020).

104. J. Orell-Miquel, F. Murgas, E. Pallé, M. Lampón, M. López-Puertas, J. Sanz-Forcada, E. Nagel, A. Kaminski, N. Casasayas-Barris, L. Nortmann, R. Luque, K. Molaverdikhani, E. Sedaghati, J. A. Caballero, P. J. Amado, G. Bergond, S. Czesla, A. P. Hatzes, T. Henning, S. Khalafinejad, D. Montes, G. Morello, A. Quirrenbach, A. Reiners, I. Ribas, A. Sánchez-López, A. Schweitzer, M. Stangret, F. Yan, M. R. Zapatero Osorio, A tentative detection of He I in the atmosphere of GJ 1214 b. *Astron. Astrophys.* **659**, 55–66 (2022).

105. D. J. M. Petit dit de la Roche, M. E. van den Ancker, P. A. Miles-Paez, An upper limit on the extended helium atmosphere of GJ 1214 b. *Res. Notes AAS* **4**, 231 (2020).

106. R. Cloutier, D. Charbonneau, D. Deming, X. Bonfils, N. Astudillo-Defru, A more precise mass for GJ 1214 b and the frequency of multiplanet systems around mid-M dwarfs. *Astron. J.* **162**, 174–188 (2021).

107. S. Vissapragada, H. A. Knutson, N. Jovanovic, C. K. Harada, A. Oklopčić, J. Eriksen, D. Mawet, M. A. Millar-Blanchaer, S. Tinyanont, G. Vasisht, Constraints on metastable helium in the atmospheres of WASP-69b and WASP-52b with ultranarrowband photometry. *Astron. J.* **159**, 278–290 (2020).

108. R. Allart, V. Bourrier, C. Lovis, D. Ehrenreich, J. Aceituno, A. Guijarro, F. Pepe, D. K. Sing, J. J. Spake, A. Wyttenbach, High-resolution confirmation of an extended helium atmosphere around WASP-107b. *Astron. Astrophys.* **623**, 58–63 (2019).

109. J. Kirk, M. K. Alam, M. López-Morales, L. Zeng, Confirmation of WASP-107b's extended helium atmosphere with keck II/NIRSPEC. *Astron. J.* **159**, 115–123 (2020).

110. T. Močnik, C. Hellier, D. R. Anderson, B. J. M. Clark, J. Southworth, Starspots on WASP-107 and pulsations of WASP-118. *Mon. Notices Royal Astron. Soc.* **469**, 1622–1629 (2017).

111. C. Piaulet, B. Benneke, R. A. Rubenzahl, A. W. Howard, E. J. Lee, D. Thorngren, R. Angus, M. Peterson, J. E. Schlieder, M. Werner, L. Kreidberg, T. Jaouni, I. J. M. Crossfield, D. R. Ciardi, E. A. Petigura, J. Livingston, C. D. Dressing, B. J. Fulton, C. Beichman, J. L. Christiansen, V. Gorjian, K. K. Hardegree-Ullman, J. Krick, E. Sinukoff, WASP-107b's density is even lower: A case study for the physics of planetary gas envelope accretion and orbital migration. *Astron. J.* **161**, 70–82 (2021).
